# Development of a Syrian hamster anti-PD-L1 monoclonal antibody enables oncolytic adenoviral immunotherapy modelling in an immunocompetent virus replication permissive setting

**DOI:** 10.3389/fimmu.2023.1060540

**Published:** 2023-02-03

**Authors:** James H. A. Clubb, Tatiana V. Kudling, Mykhailo Girych, Lyna Haybout, Santeri Pakola, Firas Hamdan, Víctor Cervera-Carrascon, Annabrita Hemmes, Susanna Grönberg-Vähä-Koskela, João Manuel Santos, Dafne C. A. Quixabeira, Saru Basnet, Camilla Heiniö, Victor Arias, Elise Jirovec, Shreyas Kaptan, Riikka Havunen, Suvi Sorsa, Abdullah Erikat, Joel Schwartz, Marjukka Anttila, Katri Aro, Tapani Viitala, Ilpo Vattulainen, Vincenzo Cerullo, Anna Kanerva, Akseli Hemminki

**Affiliations:** ^1^ Cancer Gene Therapy Group, Translational Immunology Research Program, Faculty of Medicine, University of Helsinki, Helsinki, Finland; ^2^ R&D Department, TILT Biotherapeutics Ltd, Helsinki, Finland; ^3^ Research Program Unit (RPU), University of Helsinki, Helsinki, Finland; ^4^ Department of Physics, University of Helsinki, Helsinki, Finland; ^5^ Laboratory of ImmunoViroTherapy, Faculty of Pharmacy, University of Helsinki, Helsinki, Finland; ^6^ Drug Research Program, Division of Pharmaceutical Biosciences, Faculty of Pharmacy, University of Helsinki, Helsinki, Finland; ^7^ Institute for Molecular Medicine Finland (FIMM), Helsinki Institute of Life Sciences (HiLIFE), University of Helsinki, Helsinki, Finland; ^8^ Comprehensive Cancer Centre, Helsinki University Hospital, Helsinki, Finland; ^9^ Department of Chemistry and the Randall Centre for Cell and Molecular Biophysics, King’s College London, London, United Kingdom; ^10^ Chicago Department of Oral Medicine and Diagnostic Science, University of Illinois, Chicago, IL, United States; ^11^ Pathology, Finnish Food Authority, Helsinki, Finland; ^12^ Department of Otorhinolaryngology – Head and Neck Surgery, Helsinki Head and Neck Center, Helsinki University Hospital and University of Helsinki, Helsinki, Finland; ^13^ Department of Gynecology and Obstetrics, Helsinki University Hospital and University of Helsinki, Helsinki, Finland

**Keywords:** adenovirus, oncolytic virus, immune checkpoint inhibitor, immunotherapy, Syrian hamster, PDAC, artificial intelligence, molecular simulations

## Abstract

**Introduction:**

Immune checkpoint inhibitors (ICIs) have revolutionized the treatment of cancer, but preclinical testing of hypotheses such as combination therapies has been complicated, in part due to species incompatibility issues. For example, one of few known permissive animal models for oncolytic adenoviruses is the Syrian hamster, for which an ICI, mainly an anti-PD-L1 monoclonal antibody (mAb) was not previously available. In this study, we developed an anti-Syrian hamster PD-L1 mAb to enable the evaluation of safety and efficacy, when combining anti-PD-L1 with an oncolytic adenovirus encoding tumour necrosis factor alpha (TNFα) and interleukin-2 (IL-2) (Ad5/3-E2F-D24-hTNFα-IRES-hIL-2 or TILT-123).

**Methods:**

Recombinant Syrian hamster PD-L1 was expressed and mice immunized for mAb formation using hybridoma technology. Clonal selection through binding and functional studies in vitro, in silico and in vivo identified anti-PD-L1 clone 11B12-1 as the primary mAb candidate for immunotherapy modelling. The oncolytic virus (OV) and ICI combination approach was then evaluated using 11B12-1 and TILT-123 in a Syrian hamster model of pancreatic ductal adenocarcinoma (PDAC).

**Results:**

Supernatants from hybridoma parent subclone 11B12B4 provided the highest positive PD-L1 signal, on Syrian hamster PBMCs and three cancer cell lines (HT100, HapT1 and HCPC1). In vitro co-cultures revealed superior immune modulated profiles of cell line matched HT100 tumour infiltrating lymphocytes when using subclones of 7G2, 11B12 and 12F1. Epitope binning and epitope prediction using AlphaFold2 and ColabFold revealed two distinct functional epitopes for clone 11B12-1 and 12F1-1. Treatment of Syrian hamsters bearing HapT1 tumours, with 11B12-1 induced significantly better (p<0.05) tumour growth control than isotype control by day 12. 12F1-1 did not induce significant tumour growth control. The combination of 11B12-1 with oncolytic adenovirus TILT-123 improved tumour growth control further, when compared to monotherapy (p<0.05) by day 26.

**Conclusions:**

Novel Syrian hamster anti-PD-L1 clone 11B12-1 induces tumour growth control in a hamster model of PDAC. Combining 11B12-1 with oncolytic adenovirus TILT-123 improves tumour growth control further and demonstrates good safety and toxicity profiles.

## Introduction

Syrian golden hamsters (*Mesocricetus auratus*; hereafter referred to as hamsters) have been used as an alternative to mice in many disease models (1). They have shown advantages over mice in modelling diseases including diabetes, atherosclerosis, infectious diseases, neurology and cancer (2–6). The utility of hamsters has been notable in understanding the pathogenesis of severe acute respiratory syndrome coronavirus-2 (SARS-CoV-2) and the development of vaccines and therapeutics against COVID-19 (7–16).

More relevant to the present study is that hamsters are permissive to replication of human adenovirus, and offer a complementary immunocompetent model to murine work (17–22). Additionally, we have demonstrated cross reactivity of human cytokines used in our oncolytic virus constructs including interleukin-2 (IL-2), tumour necrosis factor alpha (TNFα) and interleukin-7 (IL-7) (19, 20, 22–24). This has enabled a series of studies evaluating safety and efficacy when combining oncolytic virotherapy Ad5/3-E2F-D24-hTNFα-IRES-hIL2 (TILT-123) with adoptive cell transfer of tumour infiltrating lymphocytes (TILs) and the development of novel oncolytic viruses for cancer therapy (20, 23, 24). Another promising immunotherapy approach, is to combine oncolytic virotherapy with immune checkpoint inhibitors (ICI) (25). Unfortunately, a major caveat preventing representative preclinical evaluation of aforementioned strategy has been the lack of hamster ICIs.

The mouse model is the most commonly used preclinical animal model to evaluate the effectiveness of combinatorial immunotherapy treatment approaches (26). However, the mouse model is not permissive to replication of several human viruses and often requires mouse-adapted variants of the human therapy or humanized mice (27–30). Consequently, it is a challenge to representatively simulate the effects of critical OV features such as viral oncolysis and promoter specific replication and transgene expression in an immunocompetent setting. This has been the case in several preclinical studies, in which we used an adenovirus encoding murine IL-2 and TNFα controlled by a ubiquitously expressed cytomegalovirus (CMV) promoter, instead of the chimeric E2F promoter-driven TILT-123 which replicates and expresses transgenes only in tumour cells (31–35).

The growing use of hamsters, and the development of new immunotherapies highlights the need for hamster-specific reagents, such as *in vivo* suitable monoclonal antibodies (mAb) and for applications including immunoassays. Thus, in this present study, our aim was to develop an anti-Syrian hamster PD-L1 mAb to enable reliable (replication permissive setting) preclinical evaluation of a combination approach using an oncolytic adenovirus (TILT-123) with an ICI (anti-PD-L1).

We successfully generated novel mAbs against hamster PD-L1, demonstrating the rationale for use as both *in vitro* and *in vivo* research tools. We characterised the binding affinity and structure by *in silico* modelling of mAb clones (11B12-1 and 12F1-1). Using novel AI models and atomistic resolution molecular dynamics simulations we predict the binding poses of the mAb/PD-L1 complex and unravel the underlying structural factors behind binding of mAb clones to PD-L1. We show their utility of these mAb clones for co-cultures, enzyme-linked immunosorbent assay (ELISA), immunohistochemistry (IHC) and flow cytometry. We also demonstrated better anti-tumour response in a hamster model of pancreatic ductal adenocarcinoma (PDAC) when using 11B12-1 monotherapy vs IgG2a or PBS control. This was subsequently confirmed in a validation experiment which also revealed better tumour growth control when combining oncolytic adenovirus TILT-123 with 11B12-1 vs respective monotherapies and IgG2a control. Safety and toxicity profiles of hamsters treated with the combination therapy strategy were evaluated, as well as evidence of cross-reactivity of hamster anti-PD-L1 clones with human PD-L1. We also report the first use of fine needle tumour biopsies, enabling immunotherapy characterisation whilst reducing the use of animals in research, in the hamster model.

## Materials and methods

### Cell lines

The Syrian hamster oral cancer cell line HCPC-1 was a kind gift from Dr. Joel Schwartz (University of Illinois at Chicago, USA). The Syrian hamster HapT1 (PDAC cancer cell line) was obtained from Leibniz Institute (DSMZ, Braunschweig, Germany) and HT100 (lung adenocarcinoma cell line) was obtained from the Japanese Collection of Research Bioresources Cell Bank (Osaka, Japan). Human cell lines A549 (lung cancer), HSC-3 (oral cancer) and Panc 1 (pancreatic cancer) were all obtained from American Type Culture Collection (ATCC; LGS standards, USA). Ovarian patient derived xenograft (PDX) was developed in house and is described previously (24). All cell lines were cultured under manufacturers recommended conditions, and cultures were passaged three to four times prior to use in the experiments.

Human peripheral blood mononuclear cells (PBMC) were isolated using Lymphoprep (StemCell Technologies, Vancouver, Canada) from healthy donor whole blood obtained from the Red Cross Blood Service (Helsinki, Finland). PBMCs were washed with phosphate buffered saline (PBS) followed by 4–5 min incubation at room temperature with ACK lysis (Sigma-Aldrich, Missouri, USA) buffer to remove red blood cells; then washed again with PBS.

### Recombinant syrian hamster PD-L1 production

Production of recombinant protein, immunization, cell fusion and antibody production were performed by Genscript, USA. The Syrian hamster PD-L1 protein sequence (isoform X1) can be found using NCBI Reference Sequence - XP_005063766.1. The signal peptide and transmembrane region were removed from the sequence and C-terminal his-tag was added for expression and purification. The recombinant protein was purified by Ni-NTA affinity chromatography. Concentration was determined as 1.45 mg/ml (by BCA) and purity was ≥90% (SDS-PAGE). Final protein was stored in PBS, 0.5% Sodium Lauryl Sarcosine, 10% Glycerol, pH 7.4 at -80° C until further use.

### Immunization and selection of mouse for cell fusion

Five (#3553, 3554, 3555, 3556, and 3557) BALB/C mouse were immunised with the recombinant Syrian hamster PD-L1 protein according to the following schedule. Pre-immune serum was taken from the mice on day -4 followed by primary immunization (day 0) with 50 μg of recombinant PD-L1 per mouse, boost 1 (day 14) with 25 μg/mouse, boost 2 (day 28) with 25 μg/mouse and final boost on day 50 ± 7 days with 25 μg/mouse. Cell fusion was performed 4 days after the final boost and two test bleeds (7 days after each boost) were performed to confirm immune response in serum by indirect ELISA.

Pre-immunization and the third immunization serum was used for flow cytometric analysis of PD-L1 expression on HapT1 to confirm mouse for cell fusion. These two time points were used as we would expect to observe the most notable fold change difference (clear result for selection of mouse for cell fusion), as a result of an adaptive immune response and development of high antibody titre. HapT1 cells grown in a T175 were harvested and seeded onto a U-bottom 96 well plate at 1x10^6^ cells/well. Cells were stained with either serum or isotype control (Mouse IgG2a Isotype Control from murine myeloma, M5409; Sigma-Aldrich) at 1:100 for 1 hour in staining buffer. After washing three times, secondary antibody (Anti-Mouse IgG (Fab specific) F(ab′)2 fragment–FITC antibody produced in goat, F2653; Sigma-Aldrich) was added for 1 hour. FLA-1 fold change increase from pre-immunization to post immunisation was used to select mouse.

Two rounds of cell fusions were performed by electro-fusion. All fused cells from each cell fusion were plated into 96-well plates. Fusion was performed with mouse myeloma cell line SP2/0.

### Hybridoma sequencing

Total RNA was isolated from the hybridoma cells following the manufacturer’s instructions. RNA was then reverse-transcribed into cDNA using either isotype-specific anti-sense primers or universal primers following the technical manual of SMARTScribe Reverse Transcriptase. Variable regions of heavy (V_H_) and light (V_L_) were amplified according to the standard operating procedure (SOP) of rapid amplification of cDNA ends (RACE) of GenScript and cloned into a standard cloning vector separately. Colony PCR was performed for positive clones screening. The consensus sequences were provided.

### Epitope binning – competitive ELISA

Flat bottom 96-well plates were coated overnight with recombinant Syrian hamster PD-L1 at 1 μg/ml, 100 μl/well in PBS. Ten sub clone supernatants were co-cultured against each other to compete for recombinant protein epitopes. The next day, secondary detection antibody (Peroxidase-AffiniPure F(ab’)2 Fragment Goat Anti-Mouse IgG, Fcγ Fragment Specific (min X Hu,Bov,Hrs Sr Prot) (Jackson ImmunoResearch) was added at 1:20,000, 100 μl/well. Clones were grouped according to their specificity against ‘epitope 1’, ‘epitope 2’, ‘epitope 3’ and ‘undetermined’.

### Relative affinity ranking of hybridoma subclone supernatants – ELISA

Flat bottom 96 well plates were coated overnight with recombinant Syrian hamster PD-L1 at 1 μg/ml, 100 μl/well in PBS. The next day the coated plate was cultured with a 1:3 twelve-step serial dilution per sub clone supernatant for 24 hours. The next day the secondary detection antibody (Peroxidase-AffiniPure F(ab’)2 Fragment Goat Anti-Mouse IgG, Fcγ Fragment Specific (min X Hu,Bov,Hrs Sr Prot) (Jackson ImmunoResearch) was added at 1:20,000, 100 μl/well. The concentration and EC50 of the sub clone supernatants were determined and sub clones were ranked according to EC50 (ng/ml).

### Selection of parental clones and subclones - in-cell ELISA

Parental and subclone supernatants were validated for presence of anti-PD-L1 antibodies using In-Cell ELISA with three Syrian hamster cell lines (HCP-1, HapT1, HT100). Cells were seeded at 5000 cells per well in 96-flat bottom well plate overnight. For selection of subclones, basal and upregulated PD-L1 expression conditions were included (upregulated = stimulated 24 hours with respective recommended media mixed (1:1) with supernatants from hamster splenocytes stimulated with 1ug/ml concanavalin A (ConA) (Sigma-Aldrich)). The following day, mouse IgG (anti-PD-L1 in clone supernatants) were quantified using the In-Cell ELISA Kit, Colorimetric (662200, Invitrogen, Rockford, USA) according to the manufacturer’s instructions.

### Preparation of HT100 tumour infiltrating lymphocytes

Hamster TILs were obtained using a culturing method as described previously by our group (19).

5–6-week-old Syrian hamsters were purchased from Harlan Laboratories (Chicago, USA. Subcutaneously implanted tumours were excised when they reached approximately 1 cm in diameter. Tumours were cut into fragments of 1–3 mm^3^ diameter and placed into six-well G-rex culturing plates (Wilson Wolf, Minnesota, USA). Culture medium (TIL media) consisted of RPMI-1640 supplemented with 20% fetal bovine serum (FBS), 1% penicillin/streptomycin, 1% l-glutamine, 15 mM HEPES, 50 µM 2-mercaptoethanol, 1 mM Na-pyruvate and 6,000 IU/mL recombinant human IL-2 (rhIL-2) (PeproTech, USA). Half of the medium was renewed five days after culture initiation and every two days after that. On day ten of culture, wells with visible TILs growth were collected and pooled for *in vitro* co-culture experiments.

### HT100 cell line/TIL co-cultures with hybridoma subclone supernatants

50,000 HT100 cells adapted to TIL media (without rhIL-2) were seeded overnight in 24-well plates. To induce upregulation of PD-L1 on HT100 cells, the TIL media was mixed (1:1) with supernatants from hamster splenocytes stimulated with 1ug/ml concanavalin A (ConA) (Sigma-Aldrich). Frozen expanded HT100 TILs were thawed on the same day and rested overnight in TIL media (without rhIL-2) in a T175 placed on a rocker at 37°-C/5% CO_2_. The next day HT100 cells were co-cultured with HT100 TILs at 1:1 and 1:2 (E/T) with hybridoma sub clone supernatants (containing anti-PD-L1) at a 1:1 ratio (TIL media: supernatant). Next day, the suspension cells were collected, washed in PBS and directly used for RNA extraction.

### RT-qPCR

RNA in the suspension cells from co-culture experiments were isolated using RNeasy extraction kit (Qiagen, Hilden, Germany) according to the manufacturer’s instructions, and concentration was measured using Qubit4 Fluorometer. The purified total RNA (200 µg) was used to synthetize cDNA with High capacity cDNA Reverse Transcription kit (Thermo Fisher) according to the manufacturer’s instructions. Resulting cDNA was used for quantitative real-time PCR. Gene expression levels of a panel of T-cell activation markers (granzyme B, Perforin, CD25, CD137, Ki67, PD-1, and IFN-γ) was measured using a primer panel previously developed by our lab (24). .The results were normalized against the content of hamster gamma actin housekeeping gene cDNA and against mock (ΔΔCt). All PCR reactions were run in duplicates.

### Animal experiments

Immunocompetent male Syrian golden hamsters, 5 weeks old, (Envigo, Indiana, USA) were used for validation of antibodies as therapeutic antibodies. For initial testing of the two antibody clones (11B12-1 and 12F1-1), hamsters (n=4 per group) were engrafted on their right lower back with a single injection of 2x10^6^ HapT1 cells. Tumour growth was followed until day 5, when 5 to 6 mm diameter was reached. Animals were randomized into one of the treatment groups: PBS (control group), IgG2a (800 µg), IgG2b (800 µg), 11B12-1 (100 µg, 300 µg, and 800 µg), 12F1-1 (100 µg, 300 µg and 800 µg). A digital calliper was used to measure the tumour progression across the experimental days. Tumour volumes were calculated as (length x width^2^)/2. The tumour volume in percentage was obtained through normalization of daily tumour volumes to their respective day 0 volume. 11B12-1 and 12F1-1 were sterile filtered, and endotoxins removed for use *in vivo*. Animals received six rounds of intraperitoneal injections and were euthanized next day after last treatment. IgG2a (InVivoMab mouse IgG2a isotype control, BE0085-25MG) and IgG2b (InVivoMab mouse IgG2b isotype control, BE0086-25MG) were purchased from BioXCell (Lebanon, USA).

### Non-terminal tumour sampling procedure using fine needle aspiration

To significantly reduce animal use in our study we performed non-terminal tumour sampling as described in Ghadially, H., et al., 2021 (36). This approach was advocated given the semi-solid constitution of the HapT1 derived PDAC tumours. Fine needle aspirations were divided for analysis by flow cytometry or preserved in RNAlater (Sigma-Aldrich, Missouri, USA), and stored at −20°C until RNA extraction.

### Bulk RNA-Seq of fine needle aspiration

RNA from fine needle aspirated cells were isolated as described above. Concentrations were adjusted following measurement using Qubit 4 Flourometer and consolidated with Agilent 4200 Tapestation. Sequencing was performed by GENEWIZ (Germany) using PolyA selection and 20-30 million reads per sample. Using DESeq2, a comparison of gene expression between groups was performed. The Wald test was used to generate p-values and log2 fold changes. Genes with an adjusted p-value < 0.05 and absolute log2 fold change > 1 were called as differentially expressed genes for each comparison.

### Flow cytometry

Hamster tumours and spleens collected next day after the last treatment, were mechanically disrupted into single cell suspensions, filtered through 70μm filters and then used for downstream analysis. Samples were stained with antibodies for CD8^+^ (PE, 12-0080-82; eBioscience, San Diego, CA, USA), CD4^+^ (PE-Cyanine 7, 25-0041-82; eBioscience, San Diego, CA, USA), and MHC II^+^ cells (FITC, 11-5980-82; eBioscience, San Diego, CA, USA). NK^+^ cells were labelled with the polyclonal antibody anti-Asialo-GM1 (Alexa Fluor-488, 53-6507-80; eBioscience, San Diego, CA, USA), and macrophages and dendritic cells (Mac-2) cells with anti-Galectin 3 (PE, 12-5301-82; eBioscience, San Diego, CA, USA). For analysis of PD-L1 on Syrian hamster adherent PBMCs, freshly isolated PBMCs were cultured in flat bottom 6 well plates overnight to allow adherent cells to attach to the bottom. The next day adherent cells were collected and stained with parental hybridoma culture supernatants followed by secondary antibody staining (secondary antibody (Anti-Mouse IgG (Fab specific) F(ab′)2 fragment–FITC antibody produced in goat, F2653; Sigma-Aldrich). Cell fluorescence for all experiments was detected using BD Accuri C6 (BD Biosciences) collecting at least 50,000 events per sample.

### Histopathology analysis

Selected hamster tissues (livers, lungs, thyroid, spleens, and kidneys) and tumours collected for histopathological analysis were fixed in 10% formalin, and routinely processed and paraffin embedded. Head and neck tumour (floor of mouth, grade 2, cT3cN3bM0, stage IVB) used in the cross-reactivity study was collected from one patient undergoing surgical resection at the Helsinki University Central Hospital (Helsinki, Finland). Samples paraffin-blocks were sectioned into 4μm thickness slides and further stained with hematoxylin and eosin (H&E). 11B12-1 was used at a dilution of 1:200 and incubated for 1.5 hours at room temperature. Detection of 11B12-1 and 12F1-1 was carried out using Bright Vision goat anti-Mouse HRP: DPVM-55HR. PD-L1 (E1L3N^®^) XP^®^ Rabbit mAb #13684 (Cell Signalling Technologies) was used as a positive control for the cross-reactivity analysis. Images were generated using 3DHISTECH Panoramic 250 FLASH II digital slide scanner at Genome Biology Unit supported by HiLIFE and the Faculty of Medicine, University of Helsinki, and Biocenter Finland. A veterinarian pathologist examined the samples slides in a blind manner.

### Structural modeling with ColabFold and AlphaFold2

We explored antibody-antigen complexes using Artificial Intelligence (AI) tools. The initial coordinates (orientation and conformation) of antibody-antigen complexes were obtained using structural prediction of protein complexes with ColabFold (37) as follows. Experimentally determined sequences of Syrian hamster PD-L1 (U2076FB030-1), 11B12-1, and 12F1-1 were obtained from GenScript (see ‘hybridoma sequencing’ in materials & methods). The experimental sequences included ATG- and His-tags and required additional processing to avoid artifacts when used for *in silico* structural modeling of antibody-antigen binding. To this end, to determine which N- and C-terminal regions of the initial sequences need to be eliminated, using ColabFold with AlphaFold2-ptm (38), we first predicted the atomistic structures separately for PD-L1, 11B12-1, and 12F1-1. Next, in each structure, terminal residues with low confidence of prediction (pLDDT score < 70) (38) were selected for removal and discarded from their sequences. The resulting processed sequences were further used for structural modeling of antibody-antigen binding.

To predict the structure of PD-L1 + 11B12-1 and PD-L1 + 12F1-1 complexes, the processed sequences were used as input to the ColabFold AlphaFold2-multimer-v2 model. 11B12-1- and 12F1-1 antigen complexes were modeled separately (39). The ColabFold input contained the processed sequences of 1. PD-L1 antigen, 2. antibody heavy chain (V_H_) (different for 11B12-1 and 12F1-1), and 3. antibody light chain (V_L_) (identical for 11B12-1 and 12F1-1). ColabFold queries for each type of antibody were repeated 4 times with a different number of recycles (38) generating 5 models during each run (8 jobs produced 40 models in total). The generated models were ranked by pTM score (38). For both antibody types, the best models were generated with 24 recycles, having pTM scores of 0.609 and 0.842 for PD-L1 + 11B12-1 and PD-L1 + 12F1-1, correspondingly. The quality of the predicted models was further assessed using the Predicted Aligned Error metric (PAE) (39) (**Supplementary Figure 5**). The coordinates of the best models were used for atomistic molecular dynamics simulations.

### Atomistic molecular dynamics simulations

Atomistic molecular dynamics (MD) simulations were performed using GROMACS 2021.5 simulation package (40). CHARMM-GUI was used to generate the simulation inputs (41). The simulations comprised PD-L1 + 11B12-1 and PD-L1 + 12F1-1 complexes with coordinates obtained from rank_1 AF2/CF models. The antigen-antibody complexes were solvated in a cubic box filled with water molecules containing 150mM KCl. The simulations were carried out at 1 Bar and 310K. We used Amber ff14SB force field for the protein (42), compatible parameters for KCl (43), and TIP3P parameters for water (44). Electrostatic interactions were calculated using the Particle Mesh Ewald (PME) technique (45). 0.9 nm cut-off was used for the real-space part of PME and short-range van der Waals interactions, as set by CHARMM-GUI for Amber ff14SB and consistent with the cut-off suggested for this force field (46). Covalent bonds of the protein were constrained using LINCS algorithm (47). After equilibration of the systems under NVT conditions, the production runs were simulated in NpT ensemble with 4 fs timestep. A large timestep was obtained by utilizing heavy hydrogens reducing their oscillatory frequencies and slowing down the fastest degrees of freedom (48). Each case (PD-L1 + 11B12-1 and PD-L1 + 12F1-1 complexes) was simulated for 1 μs with 10 independent replicas, thus the total simulation time of 20 μs. For the analysis of the simulation data, an equal number of data frames (taken once per nanosecond) were selected from each simulation/repeat to avoid any statistical bias. VMD software (49) was used to visualize the protein structures.

### Analysis of MD simulation data

For all analyses, MD trajectories were subsampled at 1 ns. To assess the relaxation of the simulation ensemble, we calculated the average root-mean square deviation (RMSD) over all the replicas as a function of time. This was done by first rotationally and translationally fitting the PD-L1 and then calculating the RMSD of the bound antibody alone. This measure allowed us to quantify how stably the antibody was bound to the PD-L1 protein. When the average RMSD (averaged over the different replicas) stabilized, we interpreted the antibody to have found its binding pose. The uncertainty associated with this measure was quantified by calculating the standard error of the mean. To understand how rigidly the antibody was bound to PD-L1, we calculated the root-mean-square fluctuations (RMSF) of the PD-L1 + antibody complex for each residue. This was calculated by first determining the average structure of the PD-L1 + antibody complex and then evaluating the root mean-square deviation of the center of masses of the individual residues from the average structure. For the contact analysis performed on the data generated by the simulations, a contact was defined to form when two heavy (non-hydrogen) atoms were closer than 4.5 Å. This analysis allowed us to understand how tightly the antibody binds to PD-L1. Similarly, a hydrogen bonding analysis was also performed to evaluate the contribution of directional contacts on the antibody antigen interactions. A hydrogen bond was determined to occur if the distance between a donor heavy atom and an acceptor heavy atom was at most 3.5 Å, and if the angle between the donor hydrogen and the acceptor heavy atom was 150° - 210°.

### Evaluation of binding kinetics and affinity of 11B12-1 and 12F1-1 by Surface Plasmon Resonance (SPR)

The SPR experiments were performed using the MP-SPR Navi 220A Bionavis surface plasmon resonance instrument. Gold sensor slides (Bionavis, Tampere, Finland) were functionalized with Protein A/G as previously described (50). The experiments which were conducted at 20°C with a flow rate of 20µl/min. Syrian Hamster anti-PD-L1 (1 µg/mL) clones (11B12-1 and 12F1.1) were captured in separate flow channels on the Protein A/G sensor slides until reaching saturation. Recombinant Syrian Hamster PD-L1 (10 µg/mL in 10 mM sodium acetate, pH 4.5) were then injected at concentrations of 2.5, 5 and 10 nM. SPR sensorgrams were then fitted globally to a bivalent affinity model (TraceDrawer v1.3., Ridgeview Instruments, Uppsala, Sweden) to provide on-rate (ka), off-rate (kd), and dissociation constant (KD) values. The equilibrium dissociation constant was determined by KD =kd/ka.

### siRNA PD-L1 knockdown

To assess the specificity of the anti-PD-L1 clone’s 12F1-1 and 11B12-1, we performed RNA interference of hamster PD-L1 on the hamster cancer cell line HT100. Three different pre-designed hamster CD274 Silencer^®^ Select duplexes (siRNA ID# 555982, 555983, 555984) and 1 recommended Silencer^®^ Select negative control (Cat# 4390843) were obtained from Invitrogen. 10,000 HT100 cells were seeded in flat bottom 96-well plates overnight followed by RNA interference according to the typical transfection procedure described in Lipofectamine ^®^ RNAiMAX Reagent protocol (Invitrogen *Protocol Pub. No.MAN0007825 Rev.1.0*). After 24 hours of interference, cells were fixed for PD-L1 detection using 12F1-1 and 11B12-1 by In-Cell ELISA as described above.

### Antibody-mediated cytotoxicity study

To evaluate if 12F1-1 (mouse IgG2b) and 11B12-1 (mouse IgG2a) could induce antibody-mediated cytotoxicity (ADCC and CDC effects) with hamster immune cells, we performed *in vitro* co-cultures following a previously described protocol (34). As there are no commercially available NK isolation kits, we used freshly isolated hamster PBMCs and splenocytes as a source of immune cells. First, 10,000 HT100 were seeded in flat bottom 96-well plates overnight then fluorescently labelled with 10µM CFSE using CellTrace ™ Cell Proliferation Kit (C34554, Invitrogen) then incubated for 1 hour. Cells were then pre-incubated with either 10µg/ml of 12F1-1, 11B12-1, IgG2a, IgG2b for 1 hour before adding PBMCS or splenocytes isolated from hamsters earlier on the same day, at an E/T ratio of 1:1 or 1:2. After 24 hours the plate was analyzed using Alexa Flour 488 setting of Hidex Sense plate reader.

### SDS PAGE

SDS-PAGE analysis was performed by Genscript. Briefly, reducing and non-reducing loading buffer were added to protein sample respectively and the final concentration of protein was closed to 0.5 mg/ml. For the reducing condition, protein was heated to 100 °C for 5-10minutes. Protein samples were then centrifuged at 10000rpm for 1min and the supernatant run using a precast gel (Genscript, Cat.No. M42012) at 145V for 60mins.

### Statistical analysis

GraphPad Prism v.8.4.2 (GraphPad Software) was used for statistical analysis and graphical representation of the data. Volcano plots were generated in RStudio. Unpaired Student’s t test was used to compare 2 groups and 1-way ANOVA with Tukey’s *post hoc* test was used to compare 3 or more groups. Mixed-model analysis was performed to evaluate the tumour progression, using the transformed logarithmic normalized tumour volumes in SPSS v.25 (IBM, Chicago, IL, USA). Survival curves were generated using the Kaplan-Meyer method and the differences of 2 curves were compared using the log-rank test. *P* values < 0.05 were considered significant.

## Results

### Immunisation of mice with recombinant Syrian hamster PD-L1 and hybridoma generation

**Figure 1:**
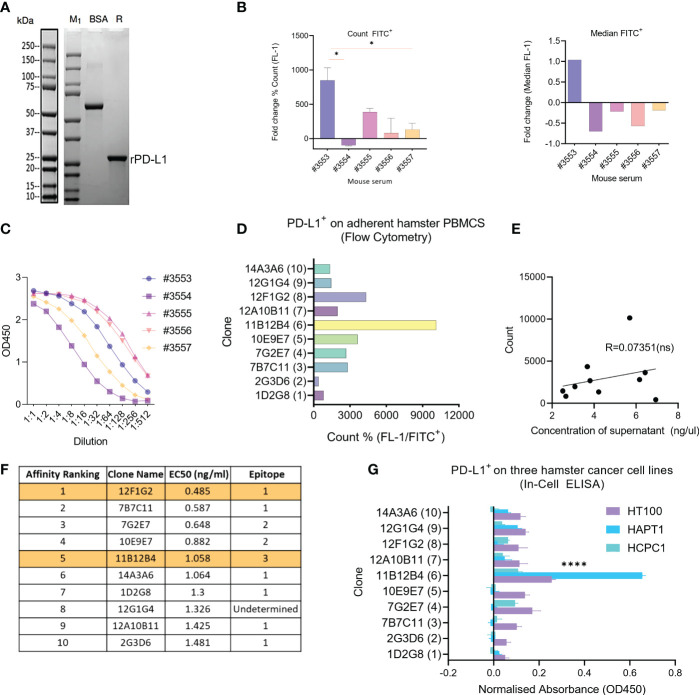
Generation of anti-Syrian hamster PD-L1 clones and their characterization for subcloning. **(A)** Confirmation of recombinant Syrian hamster PD-L1 expressed in *E.coli* by SDS-PAGE where M_1_ indicates protein ladder, BSA (2.00 µg) positive control and R: reducing conditions. Purified recombinant PD-L1 is indicated by the arrow under reducing conditions (~20-25kDa). **(B)** Detection of PD-L1 on HaPT1 cell line using antiserum of five mice immunized with recombinant PD-L1 by flow cytometry with count, mean and median (FITC^+^) shown on the left and right respectively. **(C)** ELISA results of antiserum from give mice after 3^rd^ immunization against recombinant PD-L1. **(D)** Detection of endogenous PD-L1 on adherent Syrian hamster PBMCs using anti-PD-L1 subclones from hybridoma supernatants (1:100) by flow cytometry. **(E)** Pearson’s correlation indicating a non-significant relationship between measured concentrations of hybdridoma supernatant and count used in Figure **(D)**. **(F)** Affinity ranking and epitope binning of anti-PD-L1 subclones as measured by competitive ELISA using recombinant PD-L1. The final two selected clones are highlighted in orange. **(G)** Detection of endogenous PD-L1 on three Syrian hamster cancer cell lines HT100, HapT1 and HCPC-1 using In-Cell ELISA. The absorbance values were normalized to cell number as measured by Janus Green Whole-Cell Stain. Data and error bars are presented as mean ± SEM. Statistical significance was determined by one-way ANOVA. *p < 0.05, ****p < 0.0001.

The size of the newly synthesized recombinant hamster PD-L1 protein was confirmed by SDS-PAGE showing a molecular weight of ~20kDa (**Figure 1A**). Five mice were then immunised three times with the recombinant hamster PD-L1, then pre-immunization and 3^rd^ immunization serum was compared to select a mouse for hybridoma generation. Analysis of serum by flow cytometry using HapT1 as a source of endogenous PD-L1, showed that mouse #3553 had statistically significant (p < 0.05) higher levels of anti-hamster PD-L1 antibodies compared to #3554, #3555, #3556, and #3557 as measured by % count FITC^+^ (**Figure 1B**). This was similarly shown when plotting values as median FITC^+^ where #3553 was the only mouse displaying a positive fold change (1.03) (**Figure 1B**). Levels of serum anti-PD-L1 mAbs were also measured by indirect ELISA using the recombinant protein, which showed concentration variation in order of #3555, #3556, #3553, #3557 and #3554 (from highest to lowest) (**Figure 1C**). From these data sets, mouse #3553 was selected and splenocytes were used for hybridoma development and subsequent subclone selection.

### Characterisation of anti-hamster PD-L1 clones identifies clone 11B12B4 as most promising candidate

Next, ten hybridoma clone supernatants were characterised for binding affinity, epitope binning and ability to bind to endogenous hamster PD-L1 (**Figures 1D–G**). Since PD-L1 is known to be expressed on several circulating immune cells, we isolated fresh hamster PBMCs and stained with supernatants followed by anti- mouse FITC^+^ antibody. This showed that clone 11B12B4 provided the highest % count (FITC^+^), followed by 12F1G2, 10E9E7, 7B7C11, 7G2E7, 12A10b11, 14A3A6, 12G1G4, 1D2G8 and lowest 2G3D6 (**Figure 1D**). Correlative analysis showed a non-significant relationship between the concentrations of the supernatant and the FITC^+^ count (**Figure 1E**). We also wanted to identify if the anti-PD-L1 clones could detect endogenous hamster PD-L1 on cancer cells (**Figure 1F**). We screened three hamster cancer cell lines using In-Cell ELISA, which showed a similar staining pattern to **Figure 1D**. The clone 11B12B4 provided statistically significantly higher (p < 0.0001) OD450 values compared to all other clones and was consistent across all three cell lines. Affinity ranking and epitope binning by competitive ELISA revealed 12F1G2 as having the highest binding affinity followed by 7BC11, 7G2E7, 10E19E7, 11B12B4, 14A3A6, 1D2G8, 12G1G4, 12A10B11 and lowest 2G3D6 (highest to lowest) (**Figure 1G**). Epitope binning revealed three distinct epitopes (1, 2 or 3 and one undetermined) with 6 clones binding to epitope 1, 2 clones binding to epitope 2 and 1 clone binding to epitope 3. From these data sets, 11B12B4 demonstrated most potential based on ability to detect endogenous PD-L1 and unique epitope binding (only clone binding to epitope 3). Regardless, five most promising clones (7B7C11, 7G2E7, 11B12B4, 12F1G2, 12G1G4) were selected for sub cloning and further characterisation.

### *In vitro* functional characterisation reveals immune modulating properties of subclones and supports selection of candidates for *in vivo* testing

**Figure 2:**
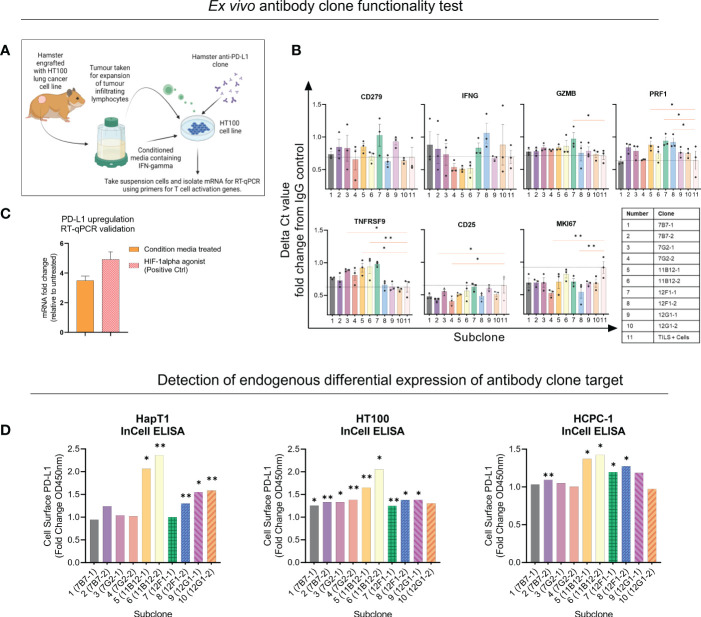
Selection of anti-Syrian hamster PD-L1 subclones by *in vitro* functionality. **(A)** Illustration of co-culture experiment design. **(B)** Seven T cell activation genes were measured from suspension cells isolated from the anti-PD-L1 subclone co-culture as measured by RT-qPCR. Values are presented as fold change from IgG control and the black dotted horizontal line represents the ‘baseline’ of HT100 + TILs without subclone. **(C)** Validating upregulation of endogenous PD-L1 on HaPT1 by RT-qPCR following exposure to conditioned media with a HIF-1 agonist as a positive control. **(D)** Detection of endogenous differential PD-L1 expression on the surface of three Syrian hamster cancer cell lines by anti-PD-L1 subclones as measured by In-Cell ELISA. Diluted conditioned media was used to upregulate PD-L1 and was compared to untreated control. PD-L1+ signal was measured by absorbance OD450 of HRP-conjugated antibody detecting mouse IgG. Clones able to detect differential expression of PD-L1 were considered more specific for PD-L1 detection. The absorbance values were normalized to cell number as measured by Janus Green Whole-Cell Stain. Data and error bars are presented as mean ± SEM. Statistical significance was determined by unpaired t-test with Welch’s correction. *p < 0.05, **p < 0.01.

Next, the five clones previously selected were subcloned into two per clone, and functional assays were performed to provide insights for selection of two candidates for *in vivo* testing. Unfortunately, there are limited commercially available research tools and cross-reactive reagents that would enable straightforward evaluation of functionality. For example, at the time of performing our experiment, there was not a commercially available hamster IFN-γ to modulate PD-L1 expression or assays to detect T cell activation related effector molecules such as IFN-γ, TNFα or granzyme B. Therefore, for the functional assays we used conditioned media from Concanavalin A stimulated hamster splenocytes as a source of IFN-γ. The first assay (co-culture) used expanded tumour infiltrating lymphocytes (TILs), matched cancer cell line (HT100) exposed to conditioned media and sub clones (**Figure 2A**). After incubating overnight, the mRNA from the suspensions cells was isolated and a panel of seven primers was used to detect expression of activation genes CD279, IFNG, GZMB, PRF1, TNFRSF9, CD25 and MKI67 with TILs and cells only used as baseline (black dotted line) (**Figure 2B**). Overall, the data shows primarily subclones of 11B12B4, 12F1G2 and 7G2E7 induce differential gene expression of activation genes compared to baseline. Subclones of 12F1G2 induced significantly higher expression of GZMB (p < 0.05), PRF1 (p < 0.05), TNFRSF9 (p < 0.001) and lower expression of MKI67 (p < 0.001). Subclones of 11B12B4 induced significantly higher expression of PRF1 (p < 0.05), TNFRSF9 (p < 0.05) whilst subclones of 7G2E7 induced significantly higher expression of TNFRSF9 (p < 0.05) and lower of CD25 (p < 0.05) and MKI67 (p < 0.001).

We also validated that our conditioned media used in the assay set up was inducing PD-L1 expression and an immunosuppressive phenotype on HT100 (**Figure 2C**). We showed that conditioned media induced a ~4 fold change increase in PD-L1 expression compared to untreated HT100 cells. This was comparable to the positive control, deferoxamine mesylate (~5-fold increase) which induces hypoxia, a physiological state known to upregulate PD-L1 expression. Next, we tested if the subclones could detect differential expression of endogenous PD-L1 on the surface of three hamster cancer cell line (HapT1, HT100, and HCPC-1). This would provide insights into functionality and specificity of the subclones, given the intrinsic role of differential PD-L1 expression on a cancer cell’s surface in regulating an immune response. We used the validated conditioned media and performed an InCell ELISA to detect differential PD-L1 expression between PD-L1^High^ and PD-L1^Low^ cells (**Figure 2D**). All subclones detected at least ~1 fold increase in PD-L1 expression, however specific subclones were more consistent than others across the three cell lines tested. Both subclones of 11B12B4 detected significantly higher fold change expression of PD-L1 on all three cell lines (p < 0.05) and consistently provided the highest PD-L1 signal compared to all other subclones. We also observed all subclones of 12F1G2 detected significantly higher expression (p < 0.05) except for subclone 12F1-1 on HaPT1. Subclones 7B7-2 was the third most consistent and detected significantly higher expression (p < 0.001) on HT100 and HCPC-1. Altogether, this functional data supported the selection and testing of subclones 12F1-1 and 11B12-1 *in vivo*.

### 11B12-1 mAb demonstrates remarkable anti-tumour efficacy in a Syrian hamster model of pancreatic ductal adenocarcinoma

**Figure 3:**
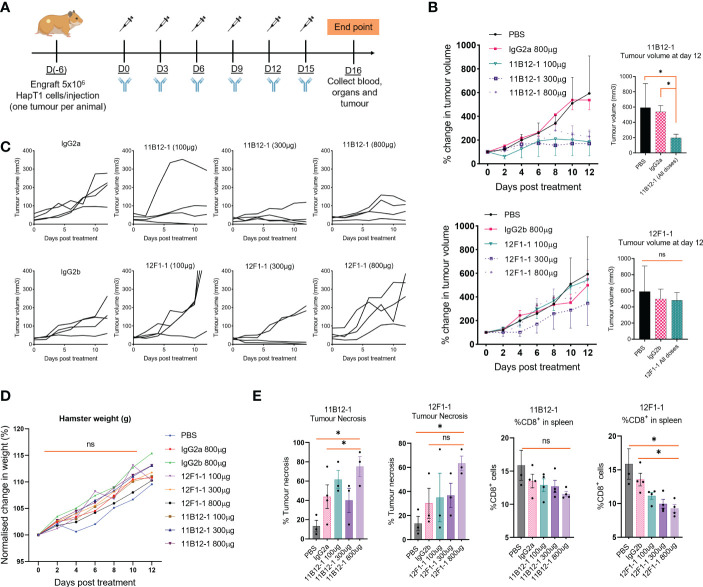
*In vivo* efficacy of anti-PD-L1 clones 11B12-1 and 12F1-1 in a Syrian hamster model of pancreatic ductal adenocarcinoma. **(A)** Schematic of treatment scheme. HapT1 (heterotopic PDAC) bearing Syrian hamsters (n=4 per group) were intraperitoneally injected with PBS (Mock), IgG isotype controls (800 µg) or Syrian hamster anti-PD-L1 (11B12-1 or 12F1-1) at three doses (100, 300 or 800 µg) every three days for a total of 6 injections. **(B)** Mean percentage change in tumour volume for 11B12-1 (upper) and 12F1-1(lower) treated hamsters and respective controls with statistical significance shown to the right. **(C)** Individual tumour growth curves. **(D)** Percentage change in weight of hamsters after treatment with anti-PD-L1 clone’s 11B12-1 and 12F1-1. **(E)** Percentage of tumour necrosis and CD8^+^ cells in spleens of hamsters as determined by histopathological analysis and flow cytometry respectively. Data is normalized to day 0. All data and error bars are presented as mean ± SEM. Statistical significance for tumour growth controls was calculated using two-way mixed model ANOVA. All other data was calculated for statistical significance using an unpaired t-test with Welch’s correction. *p < 0.05, ns not significant.

Having identified the most promising candidates, we selected 11B12-1 and 12F1-1 for large scale production and *in vivo* testing. A syngeneic hamster model of PDAC was used to test the efficacy of 11B12-1 or 12F1-1 (i.p.) at three different doses (**Figure 3A**). We observed 11B12-1 provided superior tumour growth control when compared with the isotype control (IgG2a) and PBS at all three doses tested (100 µg, 300 µg, 800 µg), with the response being dose independent (**Figure 3B** upper). The response was statistically significant when compiling the effect of all three doses of 11B12-1 (p < 0.05). In comparison, 12F1-1 did not provide better tumour growth control when compared with the isotype control (IgG2b) and PBS (**Figure 3B** lower). We did observe potential efficacy induced by 12F1-1 at 300 µg but this was not statistically significant. The individual tumour growth curves for each treatment condition can be seen in **Figure 3C**. We also identified no difference in weight change (**Figure 3D**) or serum metabolites (**Supplementary Figure 1A**) between the hamsters and their respective treatment groups suggesting that the treatments were safe and tolerable.

To provide additional insight into the functionality of the antibody clones we performed H&E stain on the treated tumours followed by histopathological examination and profiled the spleens by flow cytometry for potential changes in immune cell populations. We observed an increase (p < 0.05) in percentage tumour necrosis when comparing 11B12-1 (800 µg) to the isotype control and PBS (**Figure 3E** left). There was also an increase at 100 µg which was not statistically significant. Similarly, we observed an increase (p < 0.05) when comparing 12F1-1 (800 µg) to PBS control but not to the respective isotype control. Interestingly, we observed a non-significant increase in tumour necrosis in the isotype control compared to PBS despite similar tumour growth rate. It is known that mouse IgG2a is capable of Fc-mediated effector function and it has been described that non-specific IgG can accumulate in pro-inflammatory M1 macrophages *via* FcγRs and exacerbate inflammation (51). It is possible that the isotype control is inducing an immune reaction which is not sufficient to provide tumour growth control. When profiling the splenocytes by flow cytometry, we didn’t observe any significant changes in %CD4^+^ cells, %MHCII^+^, %GM1^+^ cells albeit a statistically significant decrease (p < 0.05) in amount of GM1+ cells in hamsters treated with 800 µg of 12F1-1 (**Supplementary Figure 1B**). Interestingly, we did observe a significant decrease (p < 0.05) in %CD8^+^ in the spleen when treating hamsters with 12F1-1 and a non-significant decrease with 11B12-1 (**Figure 3E**), both of which appeared to be dose dependent. The decrease in CD8% T cells in the spleen following treatment with 11B12-1 and 12F1-1 may be a consequence of changes in immune checkpoint signalling resulting in CD8 T cell activation and infiltration of cells away from the spleen to the tumour site. These changes will be reflected by changes in the ratio of splenic immune cells. To further examine the mechanism behind the anti-tumour efficacy observed with the two antibodies, we also performed *in vitro* antibody mediated cytotoxicity assays. We observed statistically significant cell killing when using 11B12-1 (p < 0.0001) and HT100 in the presence of hamster PBMCs at an E/T of 1:1 compared to the isotype control. We observed no significant differences when using 11B12-1 with an E/T of 2:1 or hamster splenocytes albeit a decrease in viability when using isotype control compared to 11B12-1 at E/T of 2:1. Similarly we observed a statistically significant reduction in cell viability when using 12F1-1 with PBMCs at an E/T 1:1 but not 2:1 and a significant (p < 0.001) reduction in viability when using the isotype control when compared to 12F1-1 and an E/T of 2:1.Altogether, this data demonstrates that 11B12-1 is functional as an *in vivo* mAb in terms of anti-tumour efficacy, exhibits capacity to induce ADCC and can be used to facilitate immune checkpoint blockade combination therapy studies.

### siRNA-mediated knockdown of PD-L1 on HT100 supports specificity of 12F1-1 and 11B12-1

To support our claims that 12F1-1 and 11B12-1 were specifically binding to hamster PD-L1, resulting in the *in vivo* efficacy observed, we performed RNA interference of hamster CD274 in HT100 using three different siRNAs. HT100 cells were transfected with or without siRNA and knockdown/detection analysed by InCell ELISA with either 12F1-1 or 11B12-1. **Supplementary Figures 7C, D** demonstrate differential detection of PD-L1 depending on the siRNA and antibody used for detection. When using 11B12-1 (7C) and either siRNA_1 (p < 0.05) or all three siRNA (p < 0.001) we observed statistically significant knockdown of PD-L1 on the cell surface when compared to the negative control siRNA. Interestingly, when using all three siRNA we observed almost no PD-L1 detection by 11B12-1 suggesting specificity of the antibody. However, when using siRNA_2 (p < 0.05) and siRNA_3 (ns) alone, we observed an increase in PD-L1 when compared to the negative control. The difference may be attributed to the potency of siRNA_1 compared to siRNA_2 and siRNA_3 and PD-L1 upregulation caused by an IFN mediated response to transfection. When using 12F1-1 detection we observed statistically significant knockdown when using siRNA_2 (p < 0.05) and all three siRNA (p < 0.001) when compared to negative control. However in comparison to 11B12-1 we did not observe complete reduction in PD-L1 signal when using all three siRNA. These data suggest that 11B12-1 binds more specifically to PD-L1 and supports the differences in efficacy observed *in vivo.*

### Surface Plasmon Resonance binding analysis shows 11B12-1 and 12F1-1 bind PD-L1 with high affinity

**Figure 4:**
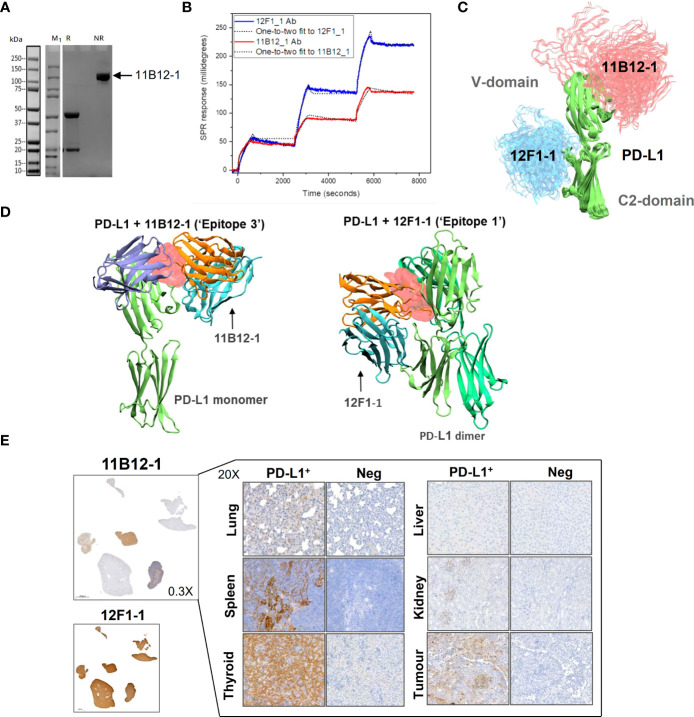
Analysis of anti-PD-L1 mAb binding characteristics supports 11B12-1 as a therapeutic antibody. **(A)** Molecular weight analysis of 11B12-1 by SDS-PAGE where M_1_ indicates protein ladder, R: reducing conditions and NR: non-reducing conditions. Purified 11B12-1 is indicated by the arrow under non-reducing conditions (~100kDa). **(B)** Sensorgrams of binding affinities of 11B12-1 and 12F1-1 with PD-L1 analyzed by surface plasmon resonance binding assay. **(C)** Snapshots of antibody-antigen complex generated by AF2/ColabFold. For each antibody 20 models from 4 independent runs are overlaid. 11B12-1 is shown in red, 12F1-1 in blue, PD-L1 is depicted in green color. All structures are aligned with respect to PD-L1 coordinates. **(D)** Upper: 11B12-1 clashes with ligand PD-1 (ice-blue). Lower: 12F1-1 clashes with PD-L1 dimer (lime & green). Steric clashes (0.3 nm cutoff) are marked as red surface. Antibody V_L_ chain is shown in orange and V_H_ chain in cyan. **(E)** Immunohistochemistry staining of Syrian hamster organs with 11B12-1 and 12F1-1. Antibody clone 11B12-1 is expanded into higher magnification (20X) for each hamster organ, whereas 12F1-1 was not because of non-specific staining.

To gain insight into the molecular mechanism linked to the anti-tumour efficacy of the antibodies observed *in vivo*, we performed analysis of binding affinity by SPR. First, we confirmed the purity and molecular weight of 11B12-1 by SDS page, which showed an approximate value of ~100 kDa (**Figure 4A**) and then validated the binding kinetics of 11B12-1 and 12F1-1 with recombinant hamster PD-L1. The association rate constant K_a_, dissociation rate constant K_d_, and equilibrium dissociation constant K_D_ of 11B12-1 and 12F1-1 are detailed in **Table 1** and the sensorgram shown in **Figure 4B**. The K_D1_ of 12F1-1 and 11B12-1 with recombinant hamster PD-L1 are 3.05 x 10^-16^ M and 1.45 x10^-16^ M respectively. The K_D2_ of 12F1-1 and 11B12-1 with recombinant hamster PD-L1 are 1.32 x 10^-5^ M and 9.27 x 10^-9^ M respectively. The Bmax1 of 12F1-1 and 11B12-1 are 310.13 and 167.60 respectively, whilst the Bmax2 are 82.69 and 23.38. The data shows that both mAbs can bind to recombinant hamster PD-L1 protein with high affinity with 11B12-1 showing stronger affinity than 12F1-1. It should be noted that the affinity values are susceptible to large variation due to the almost negligible dissociation of the two mAbs from PD-L1, as represented by the straight horizontal line in the sensograms. However, when taking into consideration the Bmax value for 12F1-1 compared to 11B12-1, we identify an agreement with the findings observed in the ELISA-based affinity ranking in **Figure 1F**.

**Table 1:** Surface Plasmon Resonance (SPR) readout for the bivalent interactions of anti-PD-L1 clones 12F1-1 and 11B12-1 with hamster PD-L1.

	12F1-1	11B12-1
B_max1_	310.13	167.60
K_a1_ (1/M x s)	2.08 x 10^12^	2.12 x 10^2^
K_d1_ (1/s)	6.34 x 10^-14^	3.08 x 10^-14^
**K_D1_ (M)**	3.05 x 10^-16^	1.45 x 10^-16^
B_max2_	82.69	23.38
K_a2_ (1/M x s)	1.10 x 10^3^	3.47 x 10^4^
K_d2_ (1/s)	1.45 x 10^-2^	3.22 x 10^-4^
**K_D2_ (M)**	1.32 x 10^-5^	9.27 x 10^-9^

### *In silico* modelling of 12F1-1 and 11B12-1 binding interactions with PD-L1 supports differences in *in vivo* anti-tumour efficacy

First, we sought to generate models which consistently predict binding epitopes of 11B12-1 and 12F1-1. Using AlphaFold2 (AF2) and ColabFold (CF) we attempted to locate binding sites of 11B12-1 and 12F1-1. These AI-based tools were not given any lead in determining a binding site or a binding pose. In all trials performed with the AI models, 11B12-1 and 12F1-1 were predicted to bind to non-overlapping regions on PD-L1 (**Figure 4C**). It was observed that 11B12-1 bound consistently (**Figure 4C**) in the vicinity of the ligand binding site in a manner that can sterically hinder the binding of the ligand (**Figure 4D** “Epitope 3”) (PDB ID: 3BIK) (52). Similarly, 12F1-1 was predicted to bind uniformly (**Figure 4C**) at the interface between V domain and C2 domain of PD-L1 (**Figure 4D** “Epitope 1”). This interface is suggested to be responsible for the dimerization of PD-L1 (PDB ID: 3FN3) (53). Thus, the predictions of the AI were found to be robust and have low statistical uncertainty. Moreover, the generated models support the observed differences in anti-tumour efficacy between 11B12-1 and 12F1-1. That is, 11B12-1 blocks the interaction between PD-lL and PD-1, whereas 12F1-1 binds to the interface of PD-L1 dimerization but does not block the PD-L1-PD-1 interaction.

### *In silico* atomistic simulations demonstrate 11B12-1 and 12F1-1 bind stably to their epitopes

To ensure that the binding poses discovered by the AI were stable, we carried out atomistic molecular dynamics simulations for the best models with the highest merit of quality in the case of each antibody as described in the methods section. In our simulations the predicted conformations remain stably bound to PD-L1 within simulation time scales in all cases. To qualify the stability of binding to PD-L1 we calculated the root-mean-square displacement (RMSD) of the antibodies with respect to the PD-L1 protein as a function of time (**Supplementary Figure 3A**). We found that within the simulation time scale the RMSD for both antibodies reached a plateau indicating equilibration of their diffusive dynamics in the vicinity of PD-L1. RMSD of 12F1-1 was found to be smaller than that of 11B12-1 by ~10Å units in the plateau region (**Supplementary Figure 3A**, last 200 ns), indicating higher stability of its binding pose. To ensure that the antibodies were stably associated with PD-L1, we compared the trajectories with the highest RMSD values of 11B12-1 and 12F1-1 (**Supplementary Videos 1**, **2**) and found that even in these worst-case scenarios the antibodies never dissociated from PD-L1 in either case. Not only does this agree with the results from SPR but also indicates that within the simulation time frame the binding pose discovered by the AI was relatively stable. To ascertain the internal fluctuations of the antibodies at the binding sites we calculated their root-mean-square fluctuations (RMSF) per residue (**Supplementary Figure 3C**) which quantifies the overall rigidity of the protein. We found that 12F1-1 has uniformly low RMSF across all residues compared to 11B12-1 while having overall smaller fluctuations in its structure. This is visualized in **Supplementary Figure 3B** where the RMSF of each residue is color-mapped on the structure of the antibody bound to PD-L1. Our analysis shows that 12F1-1, when compared to 11B12-1, binds more stably and rigidly to its epitope.

### *In silico* atomistic simulations support ELISA and SPR based binding affinity analysis

To compare and confirm the relative binding strengths of 11B12-1 and 12F1-1 with their respective epitopes we calculated the number of contacts made by the antibodies with PD-L1 as a function of time (**Supplementary Figure 4A**). As in the RMSD plot, the results support the view that the average number of contacts reaches a plateau within the simulation time frame indicating that the antibodies find a stable binding pose with PD-L1. We find that on average 12F1-1 makes 85% more contacts with PD-L1 compared to 11B12-1. To quantify the role of hydrogen bonding in these contacts we plotted their relative contribution for the two antibodies as well (**Supplementary Figure 4B**). We found that 12F1-1 makes approximately 50% more hydrogen bonds with PD-L1 compared to 11B12-1. These results propose that 12F1-1 binding to its epitope is tighter than that of 11B12-1 and are in remarkable agreement with the results of the ELISA (**Figure 1F**) and SPR (**Figure 4B**) affinity ranking.

### 11B12-1 but not 12F1-1 mAb can be used to detect hamster PD-L1 expression by immunohistochemistry

The utility of 11B12-1 and 12F1-1 to detect PD-L1 in different hamster tissues (lung, spleen, thyroid, liver, kidney and tumour [HapT1/PDAC]) was assessed by immunohistochemistry (**Figure 4E**). We first noted that 12F1-1 provided non-specific signal, evident by 100% positive signal across all tissue samples stained, thus 12F1-1 was excluded from further examination. In comparison, 11B12-1 demonstrated specificity as evident by differential expression across samples and identification of PD-L1^+^ structures within each of the stained tissues versus the negative control. Importantly we identified high PD-L1 expression in tissues that are known to also have high PD-L1 expression in humans. Primarily, we observed high expression in the lung, spleen, thyroid and tumour (54). The highest expression was observed in the thyroid which is in agreement with clinical data reporting patients develop thyroiditis after treatment with ICIs (55). Moreover, the high PD-L1 expression in the HapT1 tumour agrees with ELISA results (**Figures 1G**, **2D**) showing high PD-L1 expression on the cell line *in vitro*. Overall, the data supports the specificity of the 11B12-1 mAb and thus utility as a research tool.

### Combining 11B12-1 mAb with Ad5/3-E2F-D24-hTNFα-IRES-hIL2 is a safe and well tolerated treatment strategy

**Figure 5:**
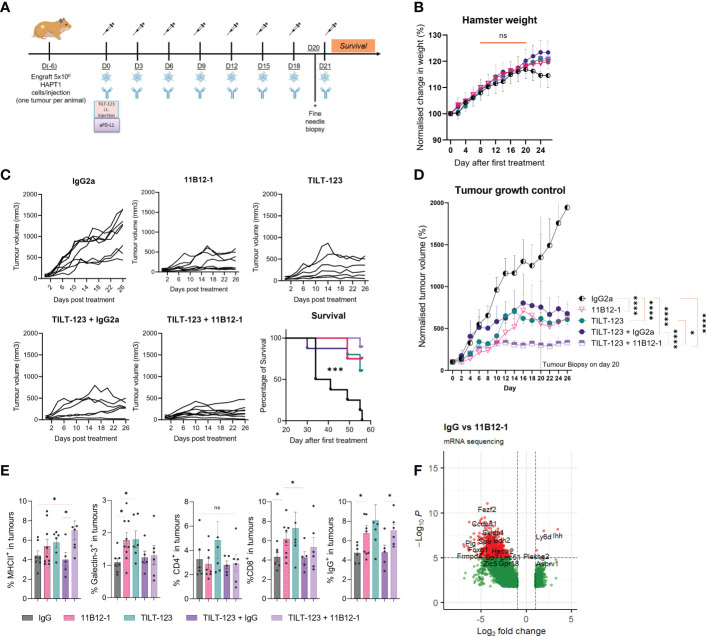
Combination therapy with 11B12-1 and oncolytic adenovirus Ad5/3-E2F-D24-hTNFα-IRES-hIL-2 provides superior tumour growth control in a Syrian hamster model of pancreatic ductal adenocarcinoma. **(A)** Schematic of treatment scheme. HapT1 (heterotopic PDAC) bearing Syrian hamsters (n=8 per group) were intraperitoneally injected with 11B12-1 (300 µg) or isotype control (300 µg) and with or without intratumoural injection of 1x10^8^ VPs of Ad5/3-E2F-D24-hTNFα-IRES-hIL-2. Treatments were given every three days for a total of 8 treatments. Fine needle tumour biopsies were taken on the day before the last treatment for evaluation of mechanism of action. **(B)** Percentage change in weight of hamsters after treatments. Data is normalized to day 0. **(C)** Individual tumour growth curves with 60 day survival analysis. **(D)** Mean percentage change in tumour volume over 28 days. **(E)** Phenotypic analysis of intratumoural immune cells in fine needle aspirates by flow cytometry. **(F)** Volcano plot for significantly differentially expressed genes between IgG2a and anti-PD-L1 clone 11B12-1. DESeq2 was used to compare gene expression between groups and the Wald test was used to generate p-values and log2 fold changes. Genes with an adjusted p-value < 0.05 and absolute log2 fold change > 1 were determined as differentially expressed genes. Significance for tumour growth controls was calculated using two-way mixed model ANOVA and survival curves by Mantel–Cox log-rank test. Statistical significance of flow cytometry data was evaluated using an unpaired t-test with Welch’s correction. *p < 0.05, ***p < 0.001, ****p < 0.0001 ns not significant. All data and error bars are presented as mean ± SEM.

Having established 11B12-1 as a functional *in vivo* anti-PD-L1 mAb, we then evaluated safety, toxicity and anti-tumour efficacy when combining 11B12-1 with an oncolytic virotherapy (Ad5/3-E2F-D24-hTNFα-IRES-hIL2). We designed the experiment so that hamsters bearing HapT1 (PDAC) tumours received 8 rounds of 11B12-1 and oncolytic virus intraperitoneally and intratumourally, respectively (**Figure 5A**). We included a fine needle biopsy at day 20 (one day before the last treatment) to evaluate transcriptomic and proteomic changes to the tumour microenvironment following treatments. First, we observed no statistically significant difference in weight between the treatment groups by day 26, albeit an approximate 2% decrease from day 18 to day 26 in hamsters treated with IgG2a (**Figure 5B**). Pathological analysis of heart, lung, kidney, thyroid and liver revealed no specific findings, although mild-moderate hydropic swelling observed more frequently in groups treated with TILT-123 (72%) or 11B12-1 (44%) compared to IgG (25%) control. These data suggest that the treatments, including the combination therapy strategy are safe and do not lead to organ toxicities.

### Combining 11B12-1 mAb with Ad5/3-E2F-D24-hTNFα-IRES-hIL2 improves tumour growth control in a Syrian hamster model of pancreatic ductal adenocarcinoma

Next, we evaluated treatment efficacy and survival benefits, which showed that 11B12-1, TILT-123, TILT-123 + IgG2a and TILT-123 + 11B12-1 provided significant better tumour growth control (p < 0.0001) and survival benefits (p < 0.001) to the hamsters when compared with the mock IgG2a control (**Figures 5C, D**). The individual tumour growth curves can be seen in **Figure 5C**. Interestingly, 11B12-1, TILT-123 and TILT-123 + IgG2a resulted in similar tumour growth control by day 26, whilst TILT-123 + 11B12-1 was significantly better when compared with TILT-123 (p < 0.05) and TILT-123 + IgG2a (p < 0.0001) but not 11B12-1 monotherapy (although a noticeable trend was observed). No differences in survival were observed between these three treatment groups by day 60. This data validates the efficacy of 11B12-1 monotherapy described in **Figure 3** and shows evidence of treatment efficacy benefits when combining TILT-123 with anti-PD-L1 mAb (11B12-1).

### Analysis of fine-needle tumour biopsies demonstrates 11B12-1 modulates the intratumoural B-cell compartment in hamster PDAC

We then analyzed the tumour biopsies taken on day 20 by flow cytometry (**Figure 5E**) and bulk RNA-Sequencing (**Figure 5F**) to gain mechanistic insight behind the observed treatment responses. Note, the selection of markers is limited due to the lack of commercially available hamster-specific or cross-reactive antibodies. Hamsters treated with 11B12-1 or TILT-123 had higher percentage (p < 0.05) of intratumoural cells expressing Galectin-3^+^, CD8b^+^ or IgG^+^ when compared with the IgG control (**Figure 5E**). The same groups also showed an increase in MHCII^+^ cells although the highest frequency (p < 0.05) was observed in the combination therapy group (TILT-123 + 11B12-1). The observed effect could be related to upregulation of PD-L1 on dendritic cells caused by uptake of virus antigen, followed by enhanced maturation and subsequent antigen presentation induced by anti-PD-L1 mAb therapy (56, 57). Indeed, we recently reported induction of tertiary lymphoid-like structures in tumours after treating with TILT-123 + anti-PD-L1 in a mouse model of SCCHN (34). Tertiary lymphoid structures being ectopic sites of local increased antigen presentation. The observed increase in intratumoural IgG^+^ (B-cells) cells may support this theory. Also, since B cell subsets express IgG, MHCII and are associated with the development and maintenance of tertiary lymphoid structures. Comparatively, we did not observe changes in percentages of intratumoural CD4^+^ cells between the groups. We also noted that the isotype control appeared to counteract TILT-123 mediated infiltration of immune cells as shown by similar values for IgG and IgG combined with TILT-123 in **Figure 5E**. This was also reflected by a non-significant reduction in tumour growth control. This may be attributed to the aforementioned non-specific induction of immune response such as engagement of Fc receptor or antibody aggregates which can activate the innate immune response. This non-specific activation can directly reduce the ability of an oncolytic adenovirus to infect, replicate, express transgenes and subsequently induce immune infiltration.

We next compared transcriptomic changes between IgG2a and 11B12-1 monotherapy treated tumours to further characterize the novel mAb and evaluate the utility of the fine-needle biopsy approach (36). Differential gene expression analysis revealed 5361 statistically significantly differentially expressed genes, with 771 upregulated and 4590 downregulated. Notably, the immunosuppressive associated gene Ido1 (IDO) was upregulated 2.17-fold (p = 0.0144) in the 11B12-1 treated group. Similarly, oncogenic genes, Myc 1.87-fold (p=0.0034), Mki67 1.81-fold (p=0.0076), Ahr 1.63-fold (p=0.0028), Erbb2 1.53-fold (p=0.0021), Kras 1.16-fold (p=0.0329) and TRIM47 1.28-fold (p=0.0491). The latter of which is known to accelerate aerobic glycolysis and tumour progression in pancreatic cancer (58). Egf, a gene profile associated with PD-L1 expression gene was downregulated 3.44-fold (p=0.0020), though CD274 was not identified as a differentially expressed gene. These data provide insight into potential anti-PD-L1 treatment resistance mechanisms. Other notable genes downregulated included Ido2 (3.05-fold) (p=0.0001) which has been reported to influence tumour progression in pancreatic cancer (59).

We also identified statistically significantly differentially expressed immune cell related genes when treating hamsters with 11B12-1. Cxcr3 (p=0.0342) was upregulated 1.6-fold, a marker primarily expressed on activated T cells (Th1 cells) and NK cells. Fcmr, a gene encoding a subunit for the Fc receptor of IgM (B cells) was upregulated 1.12-fold (p=0.0406). Conversely, Gcsam and CInk, both regulators of B-cell receptor signaling were downregulated 3.05-fold (p=0.0042) and 2.56-fold (p=0.0086) respectively. Btla and Cd22 often associated with inhibition of B-cell receptor signaling were downregulated 3.04-fold (p=0.0031) and 2.72-fold (p=0.0028) respectively. IgII5 a gene associated with naive B cells, plasma cells and activated CD4 memory T cells was downregulated 3.01-fold (p=0.0028). Ccr6 and Cxcr5, both involved in B-cell maturation and differentiation were downregulated 2.9-fold (p=0.0007) and 2.86-fold (p=0.0011) respectively. Pax5, Cr2, Cd93, Cd72, Tnfsf4, Lta, Tnfrsf13c, all regulators of B cell activation and development, were downregulated 2.6-fold (p=0.0021), 2.557-fold (p=0.0007), 2.51-fold (p=0.0031), 2.19-fold (p=0.0007), 2.19-fold (p=0.0034), 2.08-fold (0.0119) and 1.76- fold (p=0.0152) respectively. Ccr4 and Tnfrsf25 expressed in T-regs were downregulated 2.9-fold (p=0.0009) and 1.39-fold (p=0.0471) respectively, whilst Cd160, Cd226 and Tox associated with NK and T-cell function were downregulated 2.27-fold (p=0.0024), 1.36-fold (p=0.0287) and 1.23-fold (p=0.0362) respectively. IL-25, IL-34, IL-13, IL-5, IL-19, IL-11, IL-7, IL-6, IL-23a, Tgfb2, Cxcl12, Cxcl14, Cx3cr1, Ccr9 were all significantly downregulated cytokines or chemokines associated genes. Concluding, this data suggests a significant role of intratumoural B-cell immunity in the response to anti-PD-L1 therapy in hamster PDAC. Altogether, this data supports the immunomodulatory capabilities of 11B12-1 as an anti-PD-L1 mAb as well as the first reported use of fine needle biopsies in the Syrian hamster model, which supports utility to replace excessive use of animals in research.

### 11B12-1 and 12F1-1 demonstrate differential cross-reactivity with human derived cells and tissue

**Figure 6:**
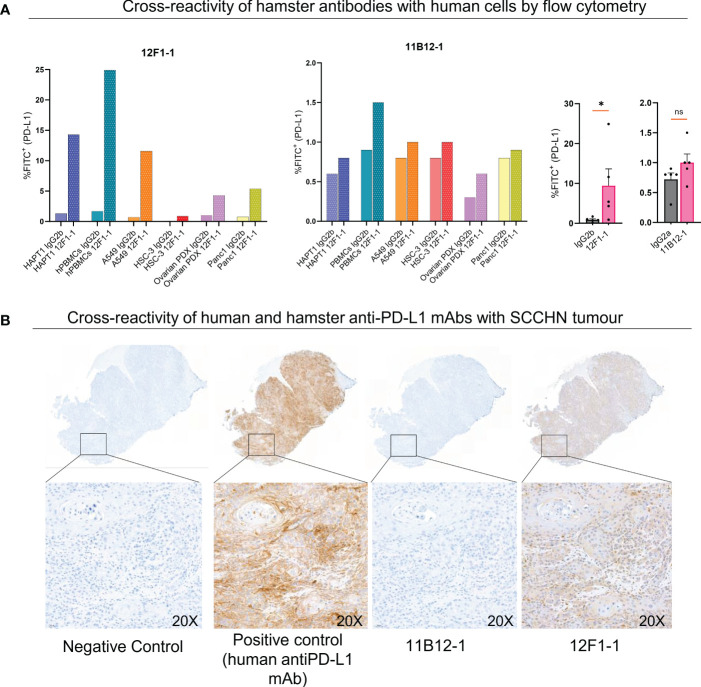
Cross-reactivity of 11B12-1 and 12F1-1 with human samples. **(A)** Evaluating cross reactivity of 11B12-1 and 12F1-1 with four human cancer cell lines (A549, HSC-3, Ovarian PDX and Panc 1), human PBMCs and hamster cancer cell like (HaptT1) by flow cytometry. Percentage of FITC^+^ was used as a readout and each stain compared with respective isotype control. **(B)** Immunohistochemistry staining of patient head and neck squamous cell carcinoma tumour (HUSHN) with 11B12-1, 12F1-1 and anti-human PD-L1 mAb (PD-L1 [E1L3N^®^] XP^®^ Rabbit mAb). Statistical significance of flow cytometry data was evaluated using an unpaired t-test with Welch’s correction. *p < 0.05; ns, not significant. Error bars are presented as mean ± SEM.

Lastly, we evaluated potential cross-reactivity of the two hamster anti-PD-L1 mAbs with human cells and tissue by flow cytometry and immunohistochemistry. For flow cytometric analysis we used HapT1 cell line as a positive control. When staining HapT1, PBMCs, A549, HSC-3, Ovarian PDX and Panc1 with 12F1-1, we observed an 11, 14.6, 16.6, 9, 4.3 and 6.75 fold change increase in %FITC^+^ (PD-L1^+^) respectively, when compared with isotype control (**Figure 6A**). In comparison when we stained with 11B12-1 we observed an 1.3, 1.6, 1.25, 1.25, 2 and 1.25 fold change increase in %FITC^+^ (PD-L1^+^). 12F1-1 was statistically significant (p < 0.05) whilst 11B12-1 was not when compiling the data from human stained cells as seen in **Figure 6A** (right). We next performed immunohistochemistry staining (**Figure 6B**) on a human tumour (squamous cell carcinoma of the head and neck). We used a validated human PD-L1 specific IHC mAb (E1L3N^®^) as a positive control. We observed ubiquitous positive PD-L1 signal when using the positive control mAb, no signal when using 11B12-1 and positive signal when staining with 12F1-1. The latter resulted in lower overall positive signal when compared with the positive control as well as differential detection in terms of structural specificity. Altogether, this data demonstrates evidence of cross-reactivity when using 12F1-1 but not 11B12-1 and therefore supports future functional evaluation. It should be noted that 12F1-1 demonstrated non-specific staining of hamster tissue by immunohistochemistry which may suggest a lack of cross reactivity of hamster anti-PD-L1 with human tissue by IHC. The differences in cross-reactivity for IHC may be caused by differences in epitope conformation following formalin cross-linking or innate cross reactivity of specific conserved binding domains. The former less likely as we observe a similar trend in signal detection for each antibody by flow cytometry using viable cells.

## Discussion

In this study, we describe the discovery and characterisation of 11B12-1 and 12F1-1, Syrian hamster mAbs that bind to hamster PD-L1 checkpoint ligand and block interactions with PD-1. This enabled tumour growth control and the subsequent evaluation of a novel immunotherapy approach using anti-PD-L1 and the oncolytic adenovirus Ad5/3-E2F-D24-hTNFα-IRES-hIL2 (TILT-123) in a virus replication permissive setting.

We utilised hybdridomas developed after immunising mice with recombinant hamster PD-L1 and performed subcloning to select two promising candidates for *in vivo* testing. Using flow cytometry and ELISA, we showed that multiple subclones were able to differentially detect and bind endogenous PD-L1 on the surface of adherent hamster PBMCs and three hamster cancer cell lines, respectively. Moreover, we detected differential surface expression of PD-L1 on the three hamster cancer cell lines following exposure to conditioned media demonstrating specificity and functionality of the subclones. Subclones 11B12-1 and 12F1-2 provided higher signal detection of PD-L1 compared to 11B12-1 and 12F1-1, which may favour the former two clones during subclone selection. However when analysing the RT-qPCR we observed higher expression of CD279 and PRF1 in 11B12-1 when compared to 11B12-2 and higher expression CD279, GZMB, TNFRS9 in 12F1-1 compared to 12F1-2. When considering functionality as a key point to consider for selection, we wanted to avoid assuming similarities between human and hamster immunology, including expression dynamics in response to anti-PD-L1 in different tumour contexts. Consequently, we considered the clearest findings provided by our experimental set-up, which also included notably downregulated genes. For example, it has been reported that CD4+ T cells downregulate IFN-y after activation in lung cancer (we used the hamster lung cancer cell line HT100) through hypermethylation of the IFNG promoter (mechanism of tumour mediated immune suppression) (60). We also observed downregulation of IFN-y when using both 11B12 subclones. This was the logic for subclone selection for mass production and subsequent experiments. The two most promising candidates selected were subclones 11B12-1 and 12F1-1, although in future studies it would be worth testing one of the two clones which bound to ‘epitope 2’ in **Figure 1F**.

From a structural perspective, we identified sequence variabilities between 12F1-1 and 11B12-1 in the heavy chain (**Supplementary Figure 2**) and two distinct epitope specificities. The *in silico* results show that the use of innovative Artificial Intelligence tools to predict the binding interface of the antibodies with PD-L1 produces statistically reliable conformations. Interestingly, the AI predicts that the 11B12-1 binds to the ligand binding site which ties in with the experimental result that epitope 3 is the ligand binding interface. Moreover, the 12F1-1 binds to a non-overlapping epitope in the AI model conforming the empirical results that it does not interfere with the ligand binding. The AI also suggests that 12F1-1 binds at the PD-L1 dimerization interface (53), providing clues to its ability to disrupt the functionality of PD-L1. We also showed, by use of SPR affinity measurements that both 12F1-1 and 11B12-1 bind strongly to hamster PD-L1 with subnanomolar bivalent affinity.

Anti-tumour efficacy of 11B12-1 and 12F1-1 was studied using *in vitro* and *in vivo* models. *In vitro* activity was analysed using PD-L1^High^ hamster lung cancer cell line HT100 and matched TIL co-cultures followed by quantification of gene expression changes associated with activation of T-cells by RT-qPCR. 11B12-1 and 12F1-1 both demonstrated ability to induce upregulation of CD279 (PD-1), IFNG (IFN-γ), GZMB (granzyme B), PRF1 (perforin) and TNFRSF9 (CD137) in the TILs following co-culture. Efficacy of 11B12-1 and 12F1-1 monotherapy *in vivo* was then tested using a hamster PDAC syngeneic model. This experiment showed that 11B12-1 but not 12F1-1 provided dose independent tumour growth control benefits and that both treatments were well tolerated in terms of safety and toxicity. We noted that tumour growth control appeared superior, when the initial tumour volume was lower at the time of treatment (It is known from the clinical that tumour burden influences response to immunotherapy), although we randomised animals as best as possible with the limited n number per group in the pilot (61). For this reason we validated the efficacy of 11B12-1 in a follow up experiment with more animals which validated anti-tumour efficacy. The difference in anti-tumour efficacy can be further explained by the *in silico* modelling and simulations which showed that 11B12-1 interferes and blocks specifically with the heterodimer PD-1-PD-L1 receptor-ligand binding domain whilst 12F1-1 with PD-L1/PD-L1 homodimerization. In general, these results indicate that the AI model reports a very robust prediction of the binding poses of the antibody/PD-L1 complex and that atomistic molecular dynamics simulations unraveled the underlying structural factors underlying the tighter binding of 12F1-1 to PD-L1.

We also examined whether the two clones could induce antibody mediated cytotoxicity (ADCC/CDC) *in vitro* to examine if the observed anti-tumour efficacy in this study was mediated by innate or adaptive immunity. We have previously shown the presence of antigen (HapT1) specific T cells in HapT1 tumours, 6 days post implantation, demonstrated by the ability to expand tumour infiltrating lymphocytes for a cell line-matched *ex vivo* cytotoxicity assay (19). This suggests that there was potential for an adaptive immune response by day 6 albeit a short time period. The ADCC assay also showed evidence of antibody mediated cytotoxicity when using hamster PBMCs with 11B12-1 and 12F1-1 but not splenocytes. The lack of efficacy when using splenocytes may be attributed to differences in cell composition, such as a lower percentage of NK cells in the spleen compared to PBMCs (62). Although this data shows evidence of antibody mediated cytotoxicity, it would be worth isolating specific cell types in future studies to confirm the cell types responsible for cell killing.

The differences in efficacy and binding off 11B12-1 and 12F1-1 were also supported by variations observed when using different target siRNA and either 11B12-1 or 12F1-1 to detect PD-L1. We observed almost complete knockdown of PD-L1 on HT100 when using all three siRNA and 11B12-1 as a detection antibody but comparatively less when using 12F1-1 albeit a significant reduction. This may suggest that 12F1-1 binds non-specifically to a similar epitope on a different cell surface protein and may support the non-specific staining observed on formalin fixed tissue. It would be worth investigating in future experiments if 11B12-1 or 12F1-1 interferes with cis-PD-L1/CD80 interaction and performing additional knockdown experiments for genes coding for proteins with similar or conserved C2-domains e.g. CD273

The anti-tumour efficacy of 11B12-1 was subsequently validated in a combination with an oncolytic adenovirus encoding IL-2 and TNFα (TILT-123). This experiment showed that 11B12-1 and TILT-123 provided tumour growth control and survival benefits to the hamsters with PDAC. Moreover, we observed that combining 11B12-1 with TILT-123 enabled superior tumour growth control benefits when compared with the monotherapies. The tumour growth control benefit was significant when comparing the combination to TILT-123 monotherapy and non-significant albeit a clear trend when comparing to 11B12-1 monotherapy. A follow up validation experiment including a longer follow up of survival and dosing might provide benefit for clinical trial treatment design. For the latter it would be more valuable to assess treatment schedule rather than dose, as we don’t expect immune checkpoint inhibitors to follow canonical dose-dependent therapeutic efficacy or adverse events as seen with chemotherapeutic drugs as shown in earlier clinical studies (63). Again, both the monotherapy and combination therapy approaches were well tolerated by the hamsters in terms of safety and toxicity profiles. We successfully performed flow cytometry and bulk-RNA-seq of tumour biopsies which showed an increase in percentage of intratumoural cells expressing MHCII, Galectin-3, CD8 or IgG. Notably, the highest percentage of cells expressing MHCII was observed in the combination therapy group and may therefore rationalise the superior tumour growth control observed when compared with monotherapies. This trend was also similarly observed with IgG^+^ cells (B-cells). This was complemented by transcriptome profiling of the biopsies, which showed upregulation of genes associated with immune checkpoint blockade resistance and immunomodulation centred on B-cell immunity, mainly downregulation. The latter may provide insight into the mechanism behind the anti-tumour efficacy and the tertiary lymphoid structure gene signature recently described by our group (34). The results may suggest that there was initial proliferation or infiltration of B cells to the tumour (as shown by flow cytometry), but were subsequently inhibited by other immunosuppressive immune cells (as shown by downregulation of B cell genes). This might explain the lack of complete responses observed with 11B12-1 monotherapy. Alternatively, it has been described that immunosuppressive B-cells highly express PD-L1 on the cell surface, and these PD-L1^hi^ subset regulate T_FH_-cell function and peripheral activation (64). Therefore, it is plausible that immune checkpoint blockade (anti-PD-L1) may relieve the immunosuppressive activity of these cell subset and enable anti-tumour activity. What was more interesting, and relevant for future vaccine studies, was the proposition that PD-L1^hi^ B-cells may direct differentiation of B-cells away from IgG producing plasma cells into memory B-cells (64). This effect has implications in vaccine efficacy and supports the rationale of testing anti-PD-L1 with vaccines. This concept can now be studied using 11B12-1 in the Syrian hamster model. Regardless, the findings suggest that modulation of B cell immunity in this tumour model is important for anti-tumour efficacy and may benefit TILT-123 efficacy and vice versa.

We also performed preliminary cross-reactivity analysis of 12F1-1 and 11B12-1 with human derived cells and tissue. This is relevant given mouse PD-L1 is able to functionally interact with human PD-1 and human anti-PD-L1 mAbs Atezolizumab and Avelumab have demonstrated cross-reactivity in mice (62). Interestingly Durvalumab is not cross-reactive in mice yet we report potential structural clashes between 11B12-1 with Durvalumab, Avelumab and Atezolizumab (**Supplementary Figure 6**). This could be partially explained by differences in species specific PD-L1 protein structure. Indeed, protein sequence comparison using Basic Local Alignment Search Tool (BLAST) showed that human PD-L1 isoform X1 (XP_047279218.1) has a 67.79% and 68.75% similarity with hamsters (XP_021080847.1) and mice (XP_030106880.1) respectively. Whilst hamsters and mice have a 74.91% similarity. The inter-species differences should be further explored to evaluate (with emphasis on functionality) human-hamster or hamster-mouse applications of 11B12-1. A study in the future using hamster ICIs with recombinant human PD-L1 would be the next step for validating cross reactivity. 11B12-1 can now serve as a species specific control for such future cross-reactive studies. This is beneficial when considering a recent report, which confirmed structural similarity of human and mouse PD-L1, whilst also emphasising differences in druggability (65).

In conclusion, 11B12-1 is the first Syrian hamster specific anti-PD-L1 mAb demonstrating functional *in vitro* and *in vivo* activity with acceptable safety and toxicity profiles. Therefore, 11B12-1 can be used for future preclinical studies including the development and characterization of immunotherapies and vaccines.

## Data availability statement

The datasets presented in this study can be found in online repositories. The names of the repository/repositories and accession number(s) can be found below: E-MTAB-12297 (Array Express).

## Ethics statement

The studies involving human participants were reviewed and approved by HUS Operatives Ethics Committee (permit numbers HUS/850/2017, HUS/3360/2019 and HUS/259/2021). The patients/participants provided their written informed consent to participate in this study. The animal study was reviewed and approved by National Animal Experiment Board (Eläinkoelautakunta, ELLA) and the Provincial Government of Southern Finland (license number ESAVI/28404/2019).

## Author contributions

JC, TK, MG, SP, FH, VC-C, AH, JMS, DQ, SB, CH, SK, RH, SS, AE, JS, IV, VC, AK, and AnnH designed the experiments. JC, TK, MG, LH, SP, FH, VC-C, AksH, SG-V-K, VA, EJ, SK, and AM conducted the experiments. JC, TK, MG, SP, FH, DQ, SK, AE, MA, IV, and AH analysed the results. All the authors contributed to writing and reviewing the manuscript. All authors contributed to the article and approved the submitted version.

## Supplementary material

Supplementary Figure 1 Analysis of serum metabolites and organs from Syrian hamster monotherapy and combination experiment reveals no significant changes after treatments. **(A)** Analysis of serum metabolites (ALAT, Crea, ALP, Urea and ASAT) was performed by the BACER core facility at the University of Helsinki (Advia, Siemens). Analysis revealed no significant difference between the treatment groups. **(B)** Analysis of hamster organs (lung, liver, kidney, heart, spleen) from the first *in vivo* experiment by a trained animal pathologist revealed no differences between the treatment groups. However there were reported reactions in the omentun likely caused by intraperitoneal injection. **(C)** Flow cytometric analysis shows changes in percentage of splenic CD4, MHCII, GM1 positive cells. Statistical significance of serum analysis data was evaluated using one-way ANOVA whilst flow cytometry with unpaired t-test with Welch’s correction.**(D)** Summary of pathological analysis of heart, lung, kidney, thymus, liver, spleen and tumour taken from hamsters in the combination experiment. *p < 0.05, ns not significant. Error bars are presented as mean ± SEM.

Supplementary Figure 2 Hybridoma sequencing results. Sequencing was performed by Genscript. Analysis of sequence variation reveals differences between **(A)** 12F1-1 and **(B)** 11B12-1 in the variable region of heavy chain.

Supplementary Figure 3
**(A)**. RMSD of antibodies. Data is presented as an average over 10 repeats for each antibody. **(B)**. RMSF of the PD-L1+antibody complex. Left. RMSF per residue of the PD-L1+11B12-1. Right. RMSF per residue of PD-L1+12F1-1. **(C)**. RMSF per residue of the antibody + antigen complex. V_H_ is the heavy chain of the antibody. V_L_ is the light chain.

Supplementary Figure 4
**(A)** Average number of contacts between heavy atoms of PD-L1 and 11B12-1/12F1-1). The average is calculated over 10 repeats as a function of time. The shaded region is the calculated standard error. **(B)** Average number of H-bonds between PD-L1 and 11B12-1/12F1-1). The average is calculated over 10 repeats as a function of time. The shaded region is the calculated standard error.

Supplementary Figure 5 PAE plots for PD-L1+11B12-1 (top) and PD-L1+12F1-1 (bottom) complexes. PAE shows predicted error (in Å) between all residue pairs (reference in Methods section). Results shown for the structures generated using ColabFold/AF2 with 24 recycles and ranked by pTM score (details in Methods section). **(A–C)** refer to PD-L1, Ab V_L_, Ab V_H_, correspondingly.

Supplementary Figure 6
*In silico* simulations predict clashes of 11B12-1 and 12F1-1 with known structures in Protein Data Bank. 12F1-1 interacts with PD-L1/PD-L1 homodimerization interface whereas 11B12-1 does not. 12F1-1 does not interact with PD-1/PD-L1 heterodimerization interface whereas 11B12-1 does. 12F1-1 does not clash with known small molecule inhibitors of PD-1/PD-L1 interface whereas 11B12-1 does. 12F1-1 does not clash with known macrolytic peptide inhibitors of PD-1/PD-L1 interface whereas 11B12-1 does. 12F1-1 demonstrates weak evidence of structural clash with monoclonal antibody Durvalumab for the PD-1/PD-L1 interface. 11B12-1 demonstrates evidence of structural clash with antibodies Durvalumab, Avelumab, Atezolizumab and a nanobody.

Supplementary Figure 7 Antibody mediated cytotoxicity of 12F1-1 and 11B12-1 and siRNA knockdown studies. **(A)** 11B12-1 and **(B)** 12F1-1 mediated cytotoxicity of CFSE stained HT100 cells using hamster PBMCs or splenocytes at an E/T ratio of either 1:1 or 2:1 after 24 hours co-culture. **(C)** 11B12-1 and **(D)** 12F1-1 mediated detection of PD-L1 on the surface of HT100 after incubation with siRNA targeting hamster PD-L1 or negative control siRNA or untreated HT100. PD-L1 was detected by absorbance (OD 450) and signal normalized to whole cell count (OD 615) and isotype control. Statistical significance of data was evaluated using an unpaired t-test with Welch’s correction. *p < 0.05, *** p < 0.001, ns not significant. All data and error bars are presented as mean ± SEM.

Supplementary Video 1 Interaction of anti-PD-L1 mAb 11B12-1 with PD-L1.

Supplementary Video 2 Interaction of anti-PD-L1 mAb 12F1-1 with PD-L1.
