# Supplementary figures and images for: Development of a Syrian hamster anti-PD-L1 monoclonal antibody enables oncolytic adenoviral immunotherapy modelling in an immunocompetent virus replication permissive setting
Source: Front Immunol. 2023 Feb 3;14:1060540. doi: 10.3389/fimmu.2023.1060540 (PMC9936529; doi:10.3389/fimmu.2023.1060540)

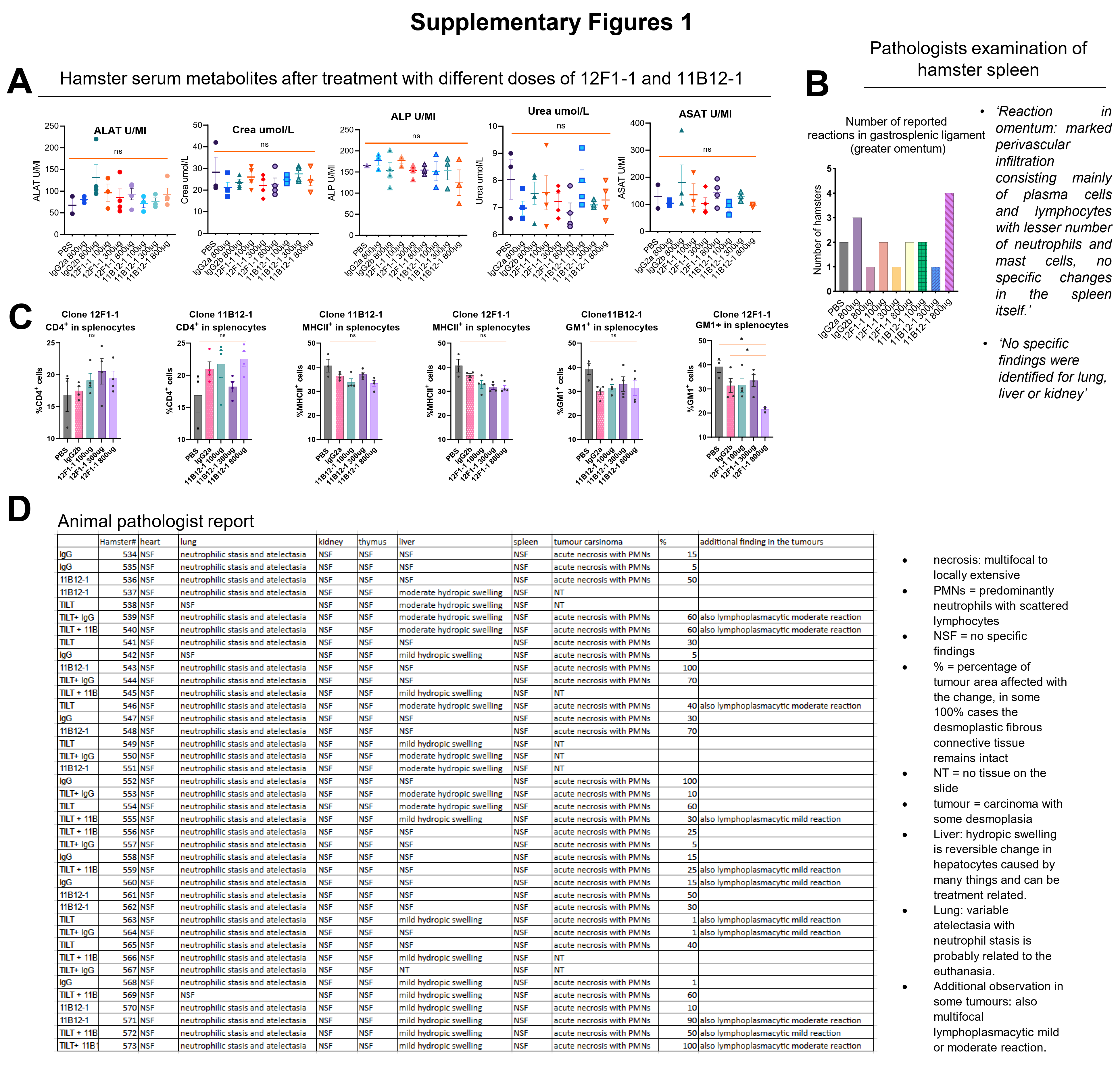

Supplement: Supplementary Figure 1 — Analysis of serum metabolites and organs from Syrian hamster monotherapy and combination experiment reveals no significant changes after treatments. (A) Analysis of serum metabolites (ALAT, Crea, ALP, Urea and ASAT) was performed by the BACER core facility at the University of Helsinki (Advia, Siemens). Analysis revealed no significant difference between the treatment groups. (B) Analysis of hamster organs (lung, liver, kidney, heart, spleen) from the first in vivo experiment by a trained animal pathologist revealed no differences between the treatment groups. However there were reported reactions in the omentun likely caused by intraperitoneal injection. (C) Flow cytometric analysis shows changes in percentage of splenic CD4, MHCII, GM1 positive cells. Statistical significance of serum analysis data was evaluated using one-way ANOVA whilst flow cytometry with unpaired t-test with Welch’s correction.(D) Summary of pathological analysis of heart, lung, kidney, thymus, liver, spleen and tumour taken from hamsters in the combination experiment. *p < 0.05, ns not significant. Error bars are presented as mean ± SEM. [file Image_1.tif]

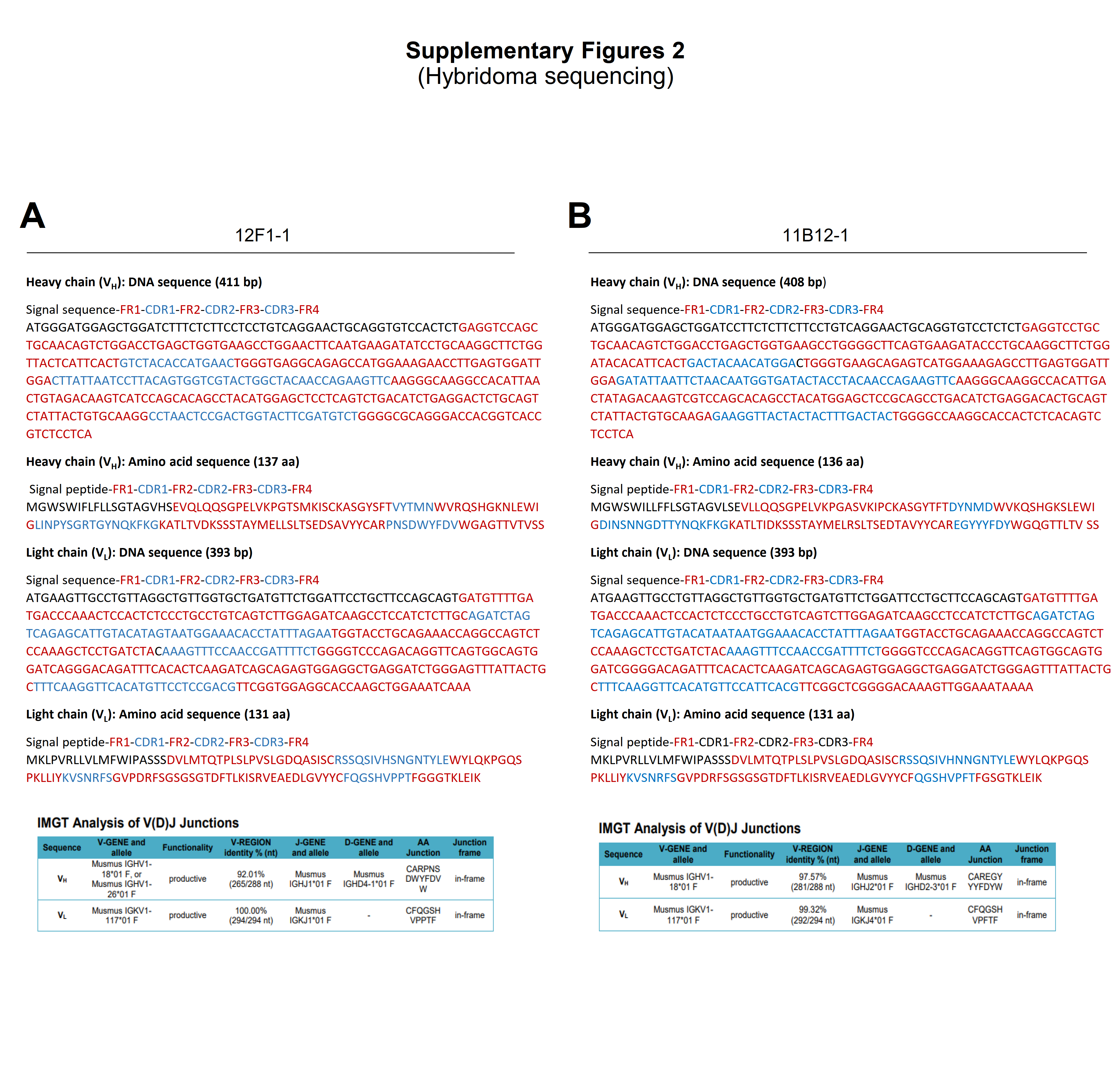

Supplement: Supplementary Figure 2 — Hybridoma sequencing results. Sequencing was performed by Genscript. Analysis of sequence variation reveals differences between (A) 12F1-1 and (B) 11B12-1 in the variable region of heavy chain. [file Image_2.tif]

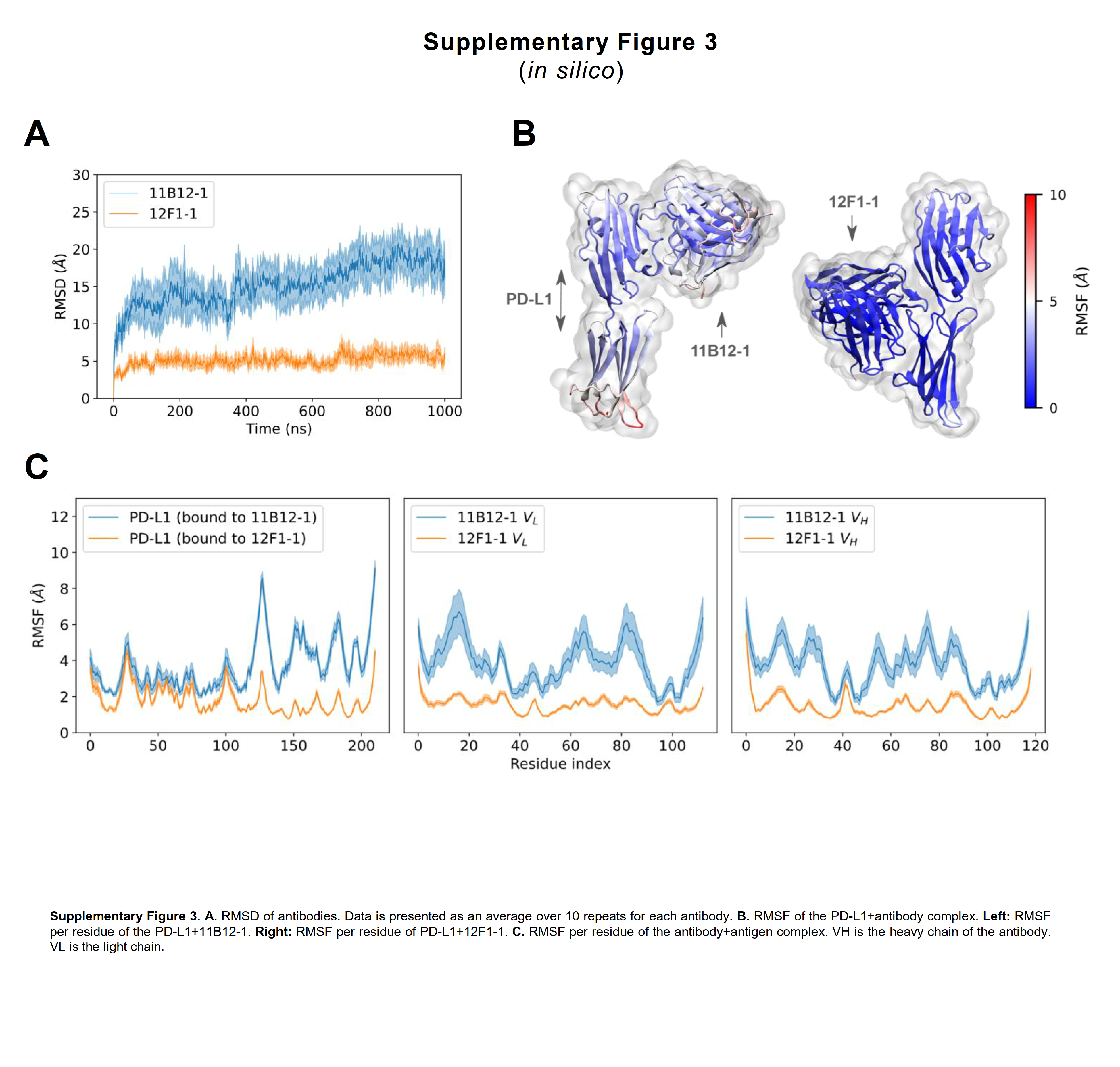

Supplement: Supplementary Figure 3 — (A). RMSD of antibodies. Data is presented as an average over 10 repeats for each antibody. (B). RMSF of the PD-L1+antibody complex. Left. RMSF per residue of the PD-L1+11B12-1. Right. RMSF per residue of PD-L1+12F1-1. (C). RMSF per residue of the antibody + antigen complex. VH is the heavy chain of the antibody. VL is the light chain. [file Image_3.tif]

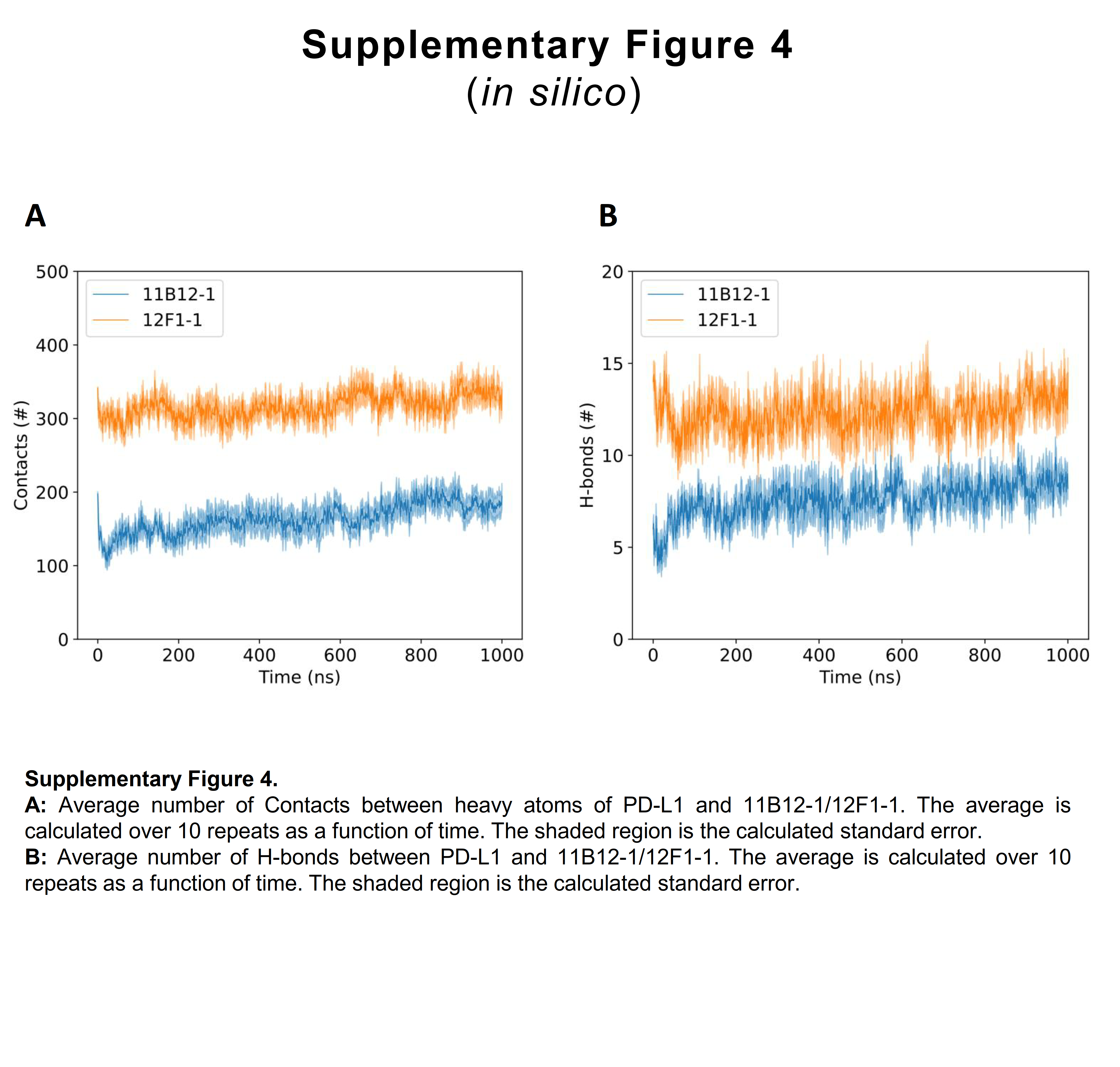

Supplement: Supplementary Figure 4 — (A) Average number of contacts between heavy atoms of PD-L1 and 11B12-1/12F1-1). The average is calculated over 10 repeats as a function of time. The shaded region is the calculated standard error. (B) Average number of H-bonds between PD-L1 and 11B12-1/12F1-1). The average is calculated over 10 repeats as a function of time. The shaded region is the calculated standard error. [file Image_4.tif]

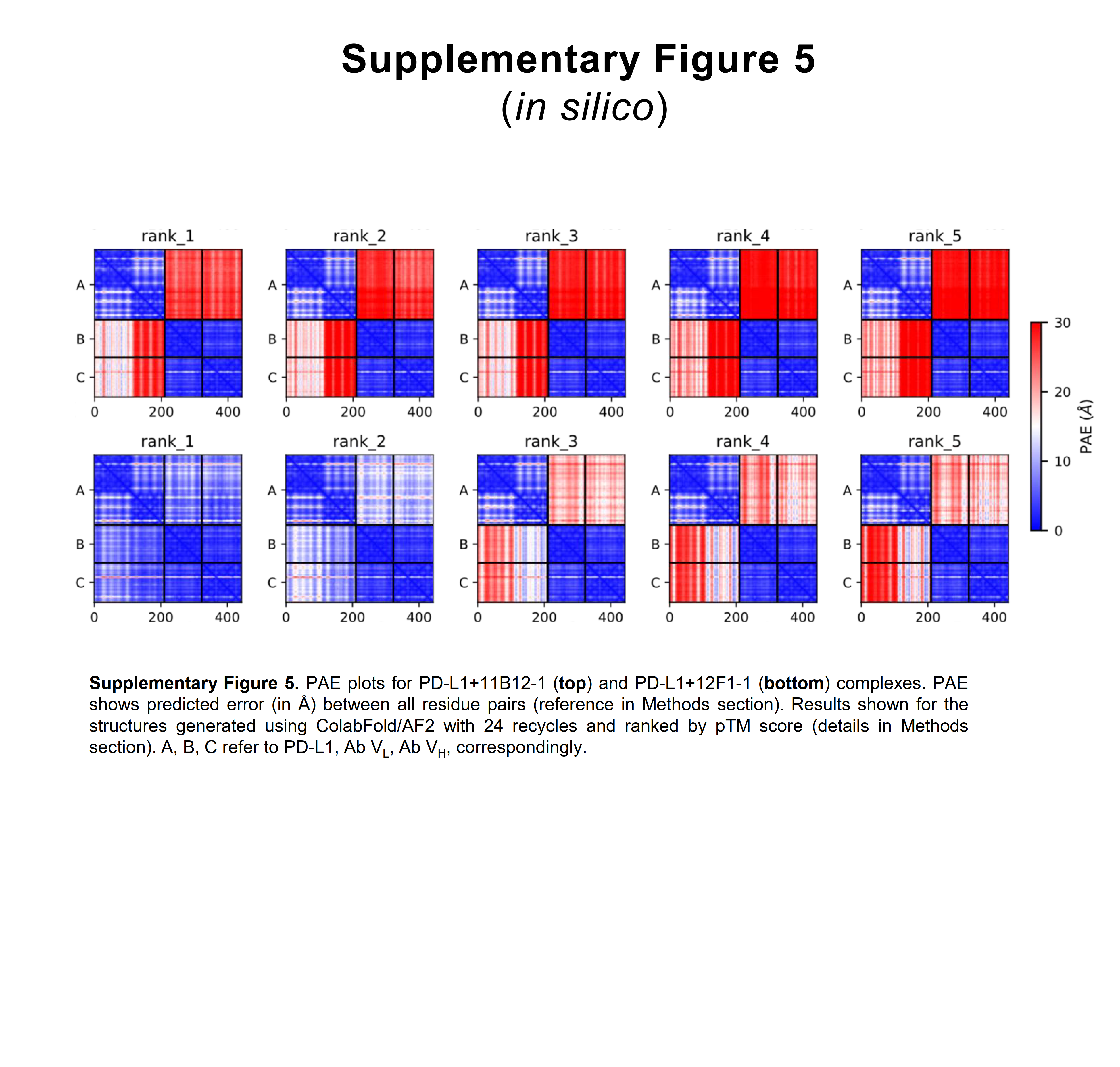

Supplement: Supplementary Figure 5 — PAE plots for PD-L1+11B12-1 (top) and PD-L1+12F1-1 (bottom) complexes. PAE shows predicted error (in Å) between all residue pairs (reference in Methods section). Results shown for the structures generated using ColabFold/AF2 with 24 recycles and ranked by pTM score (details in Methods section). (A–C) refer to PD-L1, Ab VL, Ab VH, correspondingly. [file Image_5.tif]

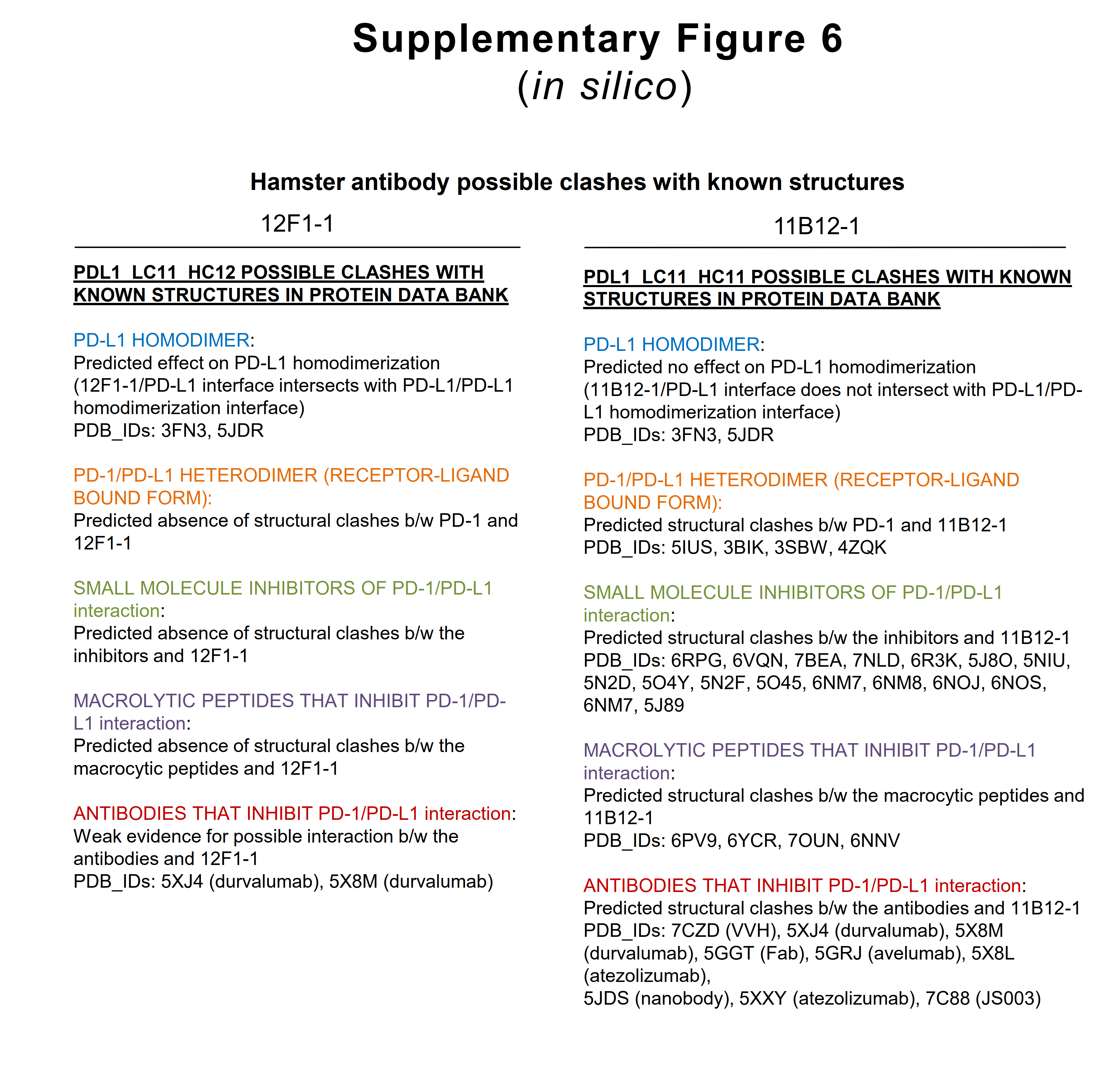

Supplement: Supplementary Figure 6 — In silico simulations predict clashes of 11B12-1 and 12F1-1 with known structures in Protein Data Bank. 12F1-1 interacts with PD-L1/PD-L1 homodimerization interface whereas 11B12-1 does not. 12F1-1 does not interact with PD-1/PD-L1 heterodimerization interface whereas 11B12-1 does. 12F1-1 does not clash with known small molecule inhibitors of PD-1/PD-L1 interface whereas 11B12-1 does. 12F1-1 does not clash with known macrolytic peptide inhibitors of PD-1/PD-L1 interface whereas 11B12-1 does. 12F1-1 demonstrates weak evidence of structural clash with monoclonal antibody Durvalumab for the PD-1/PD-L1 interface. 11B12-1 demonstrates evidence of structural clash with antibodies Durvalumab, Avelumab, Atezolizumab and a nanobody. [file Image_6.tif]

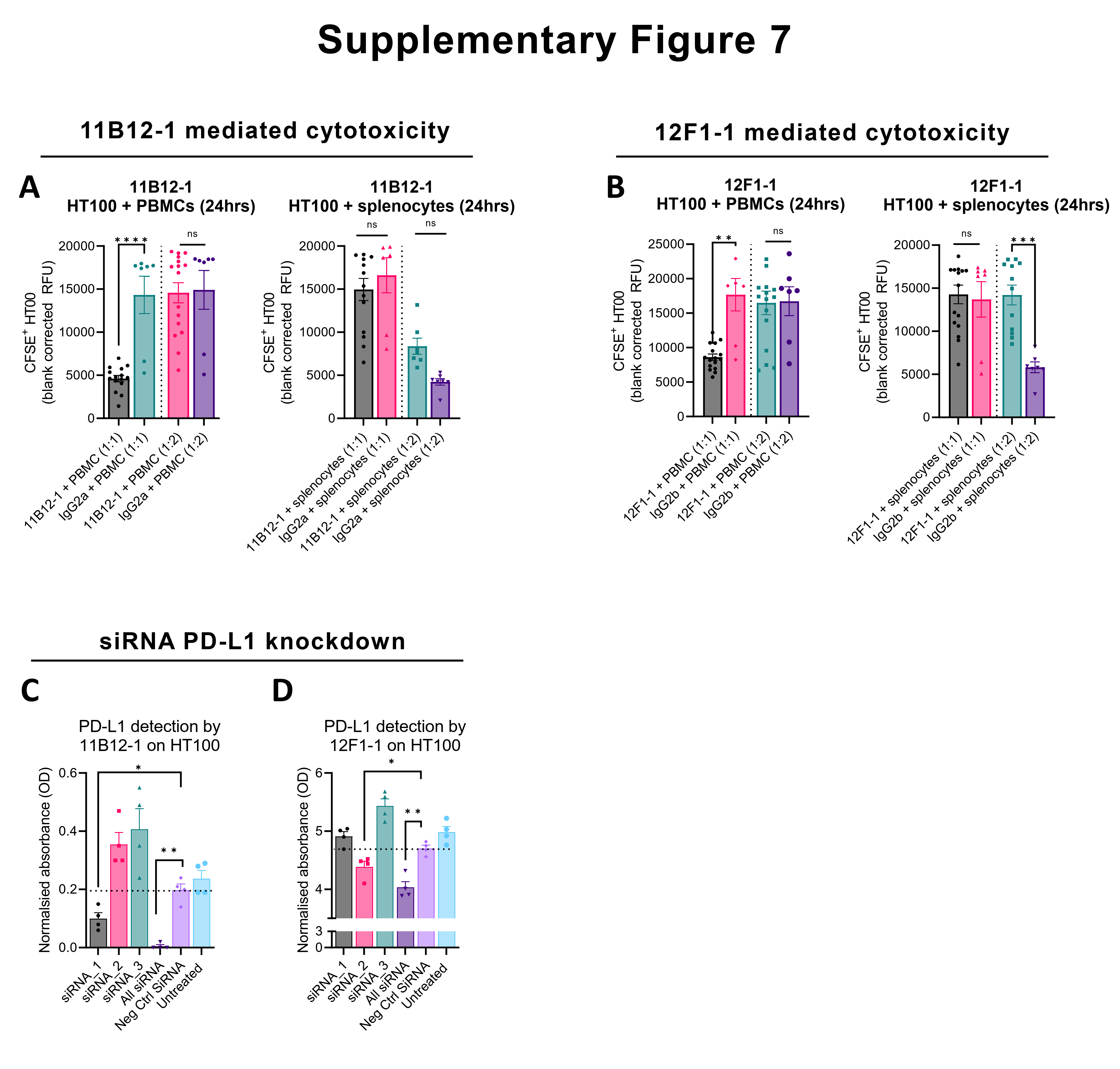

Supplement: Supplementary Figure 7 — Antibody mediated cytotoxicity of 12F1-1 and 11B12-1 and siRNA knockdown studies. (A) 11B12-1 and (B) 12F1-1 mediated cytotoxicity of CFSE stained HT100 cells using hamster PBMCs or splenocytes at an E/T ratio of either 1:1 or 2:1 after 24 hours co-culture. (C) 11B12-1 and (D) 12F1-1 mediated detection of PD-L1 on the surface of HT100 after incubation with siRNA targeting hamster PD-L1 or negative control siRNA or untreated HT100. PD-L1 was detected by absorbance (OD 450) and signal normalized to whole cell count (OD 615) and isotype control. Statistical significance of data was evaluated using an unpaired t-test with Welch’s correction. *p < 0.05, *** p < 0.001, ns not significant. All data and error bars are presented as mean ± SEM. [file Image_7.tif]
